# Renal Protection at a Metabolic Cost: A Systematic Review and Meta‐Analysis of Perioperative Use of Sodium–Glucose Cotransporter 2 Inhibitors

**DOI:** 10.1002/edm2.70180

**Published:** 2026-02-21

**Authors:** Elsayed Balbaa, Ahmed Farid Gadelmawla, Ahmed Ibrahim, AlMothana Manasrah, Ahmed Elbataa, Abdalhakim Shubietah, Mohamed S. Elgendy, Ahmed Sobhy, Ahmed Mansour, Ameer Awashra, Nourhan N. Elguindy, Mohammad Bazzazeh, Abdelhamid Ben‐Selma

**Affiliations:** ^1^ Faculty of Medicine Alexandria University Alexandria Egypt; ^2^ Faculty of Medicine Menoufia University Menoufia Egypt; ^3^ Medical Research Group of Egypt Negida Academy Arlington Massachusetts USA; ^4^ Department of Internal Medicine United Health Services—Wilson Medical Center Johnson city New York USA; ^5^ Faculty of Medicine Al‐Azhar University Cairo Egypt; ^6^ Department of Medicine Advocate Illinois Masonic Medical Center Chicago IL USA; ^7^ Faculty of Medicine Tanta University Tanta Egypt; ^8^ Faculty of Medicine Kafr Elshiekh University Kafr el‐Sheikh Egypt; ^9^ Department of Medicine An Najah National University Nablus Palestine; ^10^ Faculty of Medicine Ainshams University Cairo Egypt

**Keywords:** acute kidney injury (AKI), diabetic ketoacidosis (DKA), euglycemic ketoacidosis (eKA), meta‐analysis, perioperative care, sodium–glucose cotransporter‐2 inhibitors (SGLT2i), surgery

## Abstract

**Introduction:**

Concerns about diabetic ketoacidosis (DKA) and euglycemic ketoacidosis (eKA) are balanced against possible organ‐protective benefits in the debated perioperative management of sodium‐glucose cotransporter‐2 (SGLT2) inhibitors. This meta‐analysis compared the perioperative clinical and laboratory outcomes associated with perioperative exposure to SGLT2i.

**Methods:**

Through July 31, 2025, we searched PubMed, Web of Science, Scopus, and CENTRAL for observational studies and randomised controlled trials comparing the outcomes of preoperative use of SGLT2 inhibitors with non‐use in patients undergoing cardiac or non‐cardiac surgery. We pooled data using a random‐effects model and investigated heterogeneity using leave‐one‐out sensitivity analyses. PROSPERO‐ID: CRD420251155809.

**Results:**

There were 10 studies comprising 246,242 patients. Due to considerable heterogeneity, the primary pooled analysis revealed no significant association between SGLT2 inhibitor use and either eKA (OR 4.86; *p* = 0.11) or DKA (OR 2.21; *p* = 0.11). However, a significant increase in the risk of eKA (OR 1.11; *p* < 0.001) and DKA (OR 5.33; *p* < 0.001) was observed using leave‐one‐out sensitivity analysis to identify outliers. On the other hand, the usage of SGLT2 inhibitors was associated with a statistically significant decrease in both mortality (OR 0.73; *p* = 0.006) and acute renal injury (OR 0.68; *p* < 0.0001). The SGLT2 inhibitor group had significantly lower perioperative pH, base excess, and blood glucose levels.

**Conclusion:**

The use of perioperative SGLT2 inhibitors poses a clinical paradox between significant renoprotection and survival advantages and a latent risk of ketoacidosis concealed by considerable heterogeneity. While metabolic monitoring is essential, current surgeries requiring more prolonged withholding may need to weigh metabolic risk against the drug's significant benefit in reducing acute kidney injury and mortality.

## Graphical abstract

Across 246,542 surgical patients, perioperative SGLT2 inhibitor use was associated with a lower odds of acute kidney injury and mortality. In contrast, ketoacidosis outcomes (DKA and euglycemic ketoacidosis) showed no statistically significant difference overall, but appeared more frequent in selected sensitivity and subgroup analyses, consistent with an euglycemic masking effect. These findings underscore a perioperative paradox requiring individualised risk stratification and vigilant metabolic monitoring.

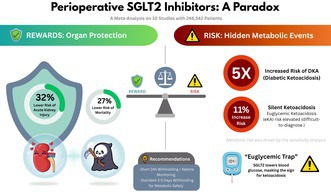

**Abbreviations**
AACE: American association of clinical endocrinology
ACC: American college of cardiology
ADA: American diabetes association
AHA: American heart association
BMI: body mass index
CABG: coronary artery bypass graft
CI: confidence interval
CPOC: centre for perioperative care
DKA: diabetic ketoacidosis
DL: DerSimonian–Laird
euDKA: euglycemic diabetic ketoacidosis
FDA: food and drug administration
*I* ^2^
: *I*‐squared (measure of heterogeneity)
ICU: intensive care unit
MD: mean difference
PaCO_2_
: partial pressure of carbon dioxide in arterial blood
pH: potential of hydrogen (measure of acidity/alkalinity)
PRISMA: preferred reporting items for systematic reviews and meta‐analyses
PROSPERO: international prospective register of systematic reviews
RCT: randomised controlled trial
ROB‐2: risk of bias 2 tool
ROBINS‐I: risk of bias in non‐randomised studies of interventions
RR: risk ratio
SGLT2i: sodium–glucose cotransporter‐2 inhibitor
UK: United Kingdom
USA: United States of America
WoS: web of science

## 1 Introduction

The United States Food and Drug Administration (FDA) has approved sodium–glucose cotransporter‐2 inhibitors (SGLT2i), also known as gliflozins, for the treatment of type 2 diabetes, recognising their role as effective antidiabetic agents [1]. SGLT2i have demonstrated efficacy in enhancing clinical outcomes in patients with heart failure, coronary artery disease, and chronic kidney disease [1, 2, 3, 4, 5].

Despite their benefits, SGLT2i have been associated with euglycemic ketoacidosis (eKA). In this rare but life‐threatening complication, patients exhibited only mildly increased blood glucose concentrations, as warned by the FDA [6, 7]. Unlike classic diabetic ketoacidosis (DKA), eKA features severe metabolic acidosis with near‐normal glucose levels, which often masks diagnosis [8]. The pathophysiology is multifactorial but primarily results from a drug‐induced imbalance in the glucagon‐to‐insulin ratio, which increases lipolysis and subsequently stimulates hepatic ketogenesis even in the fed state [9, 10, 11].

EKA is more likely to occur in the perioperative setting, where factors such as prolonged fasting [12], surgical stress, and concurrent illness increase metabolic demands and insulin requirements [13, 14]. To mitigate this, the US FDA updated its prescribing information in 2022, revising labels of SGLT2i to advise discontinuing it at least 72 h before surgery [7]. Although early case series and pharmacokinetic concepts served as the foundation for this guidance, subsequent large‐scale data have revealed a more complex clinical picture [15, 16, 17, 18]. For instance, an extensive population‐based study found that SGLT2i users had a sixfold increase in postoperative DKA, whereas 30‐day mortality decreased significantly [19]. On the other hand, Dixit et al. [19] reported that those patients who underwent emergency surgery and were unable to withhold SGLT2i did not have a significantly increased risk of DKA. Additionally, new information from the MERCURI‐2 trial [20] and the Tallarico et al. cohort [21] indicates that strict withdrawal may unintentionally deprive patients of renal protection during the delicate perioperative window. To address this knowledge gap, we conducted a systematic review and meta‐analysis to evaluate perioperative clinical and laboratory outcomes in patients using SGLT2i versus non‐users.

## 2 Methodology

### 2.1 Protocol Registration

We conducted this systematic review and meta‐analysis in accordance with the recommendations of the Cochrane Handbook of Systematic Reviews and Meta‐Analyses of Interventions and in full adherence to the Preferred Reporting Items for Systematic Reviews and Meta‐Analyses (PRISMA) (Table S1) [22, 23]. The protocol of this study was registered at PROSPERO (CRD420251155809).

### 2.2 Data Sources and Search Strategy

We conducted a comprehensive search of four electronic databases: PubMed (MEDLINE), Web of Science, Scopus, and the Cochrane Central Register of Controlled Trials (CENTRAL), from their inception through 31 July 2025. We also activated real‐time alerts in PubMed and the other databases to notify us of any newly published studies matching our predefined search strategy. Furthermore, we conducted a manual search, restricted to 2025, before the initial submission to ensure that no relevant studies were missed. The detailed search strategy for each database is presented in Table S2.

### 2.3 Eligibility Criteria

We included all studies that compare clinical and/or laboratory outcomes using the PICOS framework:

- **Population (P)**: Adult patients (aged 18 years and older) with or without diabetes mellitus undergoing any surgery, whether cardiac or non‐cardiac, and whether the procedure is emergency or elective.
- **Intervention (I)**: Preoperative use of SGLT2i.
- **Comparator (C)**: Patients not exposed to SGLT2i, typically those receiving standard care or other non‐SGLT2i antidiabetic agents.
- **Outcomes (O)**: Primary outcomes: (eKA, DKA, metabolic acidosis, and AKI). Secondary outcomes included mortality, intensive care unit (ICU) length of stay, adverse effects, including postoperative surgical‐related infection, urinary tract infection (UTI), pneumonia, atrial fibrillation, and stroke, and laboratory measures including perioperative levels of serum lactate, partial pressure of carbon dioxide (PaCO2), pH, base excess, serum potassium (K), serum sodium (Na), and blood glucose.
- **Study Design (S)**: Randomised Clinical Trial (RCT) and observational cohort or case–control studies, published in peer‐reviewed journals.

We excluded paediatric studies, single‐arm studies, case reports, case series, reviews and editorials.

### 2.4 Study Selection

The literature search results were collected and added to EndNote (Clarivate Analytics, PA, USA), where duplicates were removed. Subsequently, the results were exported to the Rayyan web [24], where two authors (A.M. and M.B.) independently conducted the screening. Screening was initially performed based on titles and abstracts, followed by a review of the full texts of the articles, in accordance with our pre‐specified eligibility criteria. Any disagreements were resolved through discussion and by consulting the senior author (A.B.). Finally, a manual review of forward and backward citations was conducted for all cited references in the included studies.

### 2.5 Data Extraction

Two authors (N.N.E. and A.E.) independently extracted the summary and baseline characteristics from the eligible studies. The summary data included the study design, the country in which the studies were conducted, recruitment period, sample size, study arms, inclusion criteria (including age in years, diabetes mellitus status, surgery type, and treatment use), primary outcomes, and conclusions. The baseline variables were patients' age in years, the number of male patients, body mass index (BMI), comorbidities rates, and surgical specialty. Any conflicts were resolved through discussion and consultation with a senior author (A.B.).

### 2.6 Risk of Bias

Two authors (A.S.E. and A.S.) independently evaluated the quality of RCTs using the Cochrane Risk of Bias 2 tool (ROB‐2) [25]. In contrast, non‐randomised studies, including cohorts, were evaluated using the Cochrane Risk of Bias in Non‐randomised Studies of Interventions (ROBINS‐I) tool [26]. Any conflicts between the authors were resolved through discussion and consultation with a senior author (A.B.).

### 2.7 Statistical Analysis

We used R version 4.3 and the meta packages for statistical analysis. We pooled results using mean differences (MD) for continuous outcomes and odds ratios (OR) for dichotomous outcomes. All effect estimates were reported with 95% confidence intervals (CI). We applied a prespecified random effects model (DerSimonian–Laird method) to account for anticipated between‐study heterogeneity. We tested heterogeneity using the chi‐square test and quantified it with the I‐squared statistic, which expresses the percentage of the total variation attributable to heterogeneity. Heterogeneity was considered statistically significant if the chi‐square (*χ* ^2^) test *p*‐value was less than 0.1. Heterogeneity was assessed according to the Cochrane Handbook [22], with *I* ^2^ values of 0%–30% indicating minimal heterogeneity, 30%–50% indicating moderate heterogeneity, 50%–75% representing substantial heterogeneity and 75%–100% signifying considerable heterogeneity. To assess the robustness of our findings, we performed a leave‐one‐out sensitivity analysis, sequentially omitting individual studies to evaluate the stability and consistency of the effect estimates [27, 28]. In addition, to explore potential sources of clinical heterogeneity, we conducted further sensitivity analyses whenever possible, focusing specifically on (1) cardiac surgeries and (2) non‐emergency elective surgeries. Test for publication bias using the Eggers test for funnel plot asymmetry was not feasible because we included fewer than 10 studies in each comparison [29].

## 3 Results

### 3.1 Search Results and Study Selection

**FIGURE 1:**
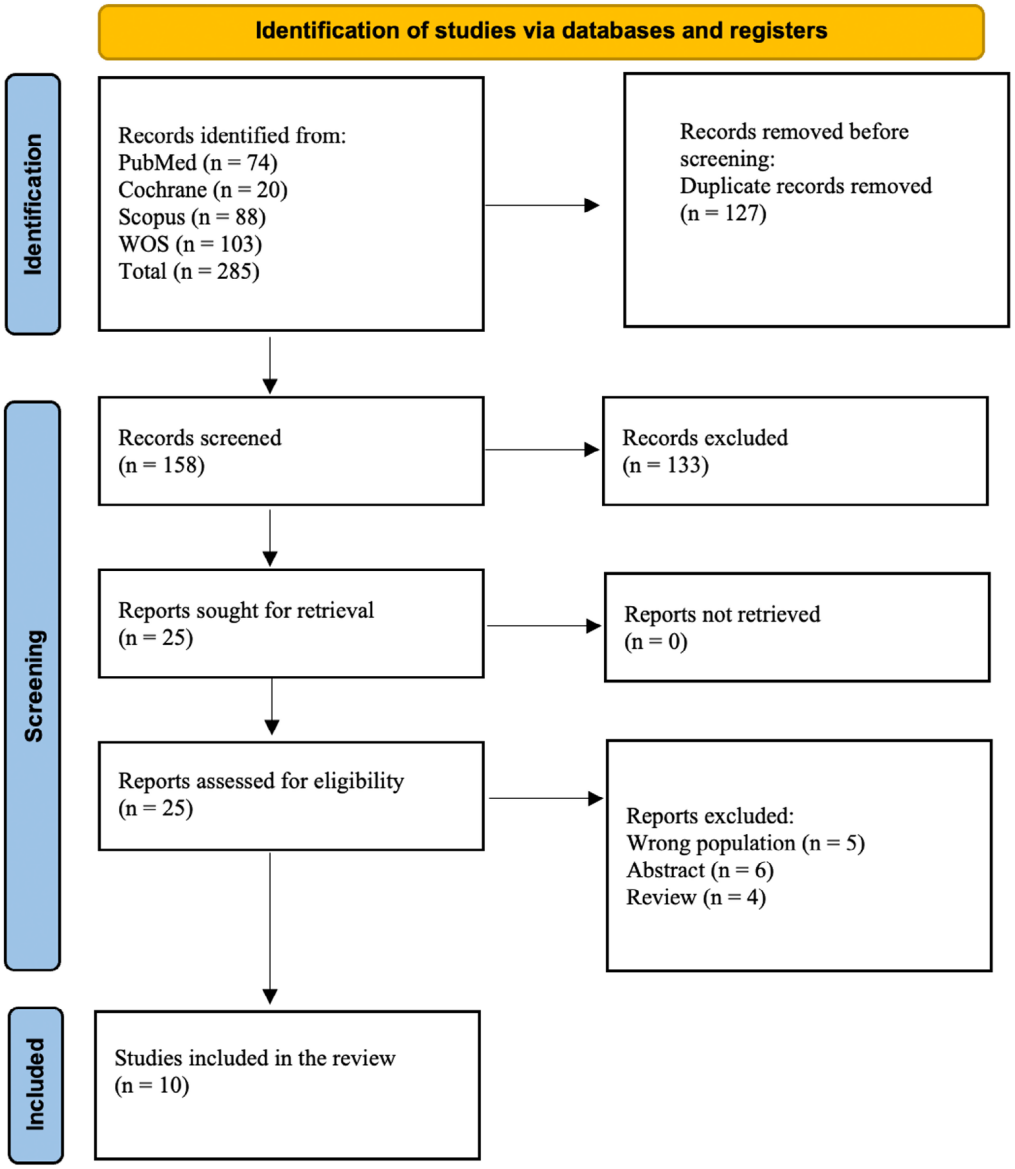
PRISMA flow diagram showing identification, screening, eligibility assessment, and inclusion of studies in the meta‐analysis.

Our initial search yielded 285 potentially relevant articles, as shown in the PRISMA diagram (Figure 1). After removal of 127 duplicate records, 158 unique records were screened by title and abstract, and 25 were selected for full‐text evaluation. After exclusion of irrelevant studies, 10 eligible articles met all inclusion criteria and were included in the analysis [6, 19, 20, 21, 30, 31, 32, 33, 34, 35].

### 3.2 Characteristics of Included Studies

Two RCTs [20, 31] and eight retrospective observational studies [6, 19, 21, 30, 32, 33, 34, 35] were published between 2022 and 2025, encompassing 246,542 patients, with 15,114 (6.1%) in the SGLT2i‐use arm and 231,428 (93.9%) in the no‐SGLT2i arm; the weighted mean age was 69.1 ± 14.8 years. Patients were predominantly male (150,121; 60.9%), with a mean BMI of 26.7 ± 3.5 kg/m2. Our population encompassed 236,069 diabetic patients (95.8%). Five studies included exclusively patients with diabetes mellitus [6, 19, 31, 33, 34], whereas the other studies included mixed populations [20, 21, 30, 32, 35]. Three studies were multicenter [19, 21, 34], whereas the remaining studies were single‐center. Preoperative management of SGLT2 inhibitors varied significantly across the included studies, with withholding periods ranging from continued administration (0 h) to cessation 5 days prior (Table 1). Studies were conducted in the United States [21, 30, 32, 34, 35], Norway [32], China [19], Japan [6], Brazil [19] and the Netherlands [20]. Five studies enrolled exclusively cardiac surgical patients [16, 17, 27, 28, 31]; 2 studies investigated non‐cardiac surgical patients [5, 29]; and three studies investigated a mixed surgical population [19, 21, 35]. Six studies enrolled exclusively patients undergoing elective surgery [6, 20, 30, 31, 32, 33], one included exclusively those undergoing emergency surgery [34], and 3 included mixed surgical populations [19, 21, 35]. Tables 1 and 2 present additional details on the characteristics of the included studies and the baseline data.

**TABLE 1:** Summary characteristics of the included studies.

Study ID	Study design	Country	Intervention/exposure	Control	SGLT2i stop	Age (Y)	DM status	Surgery type (cardiac vs. non‐cardiac)	Surgery type (cardiac vs. non‐cardiac)	eKA definition	Primary outcome	Conclusions
Tallarico 2025	Retro case‐ctrl	USA	Long‐term SGLT2i use (> 3 outpatient prescription fills without a gap of 180 days or more)	No use	No stop	18 and older	N/M	Mixed (~90% non‐cardiac, ~10% cardiac)	Mixed (~91.5% elective, ~8.5% emergency)	Defined by strict laboratory cut‐offs (pH < 7.3 and bicarbonate < 18 mEq/L) in the presence of normoglycemia (< 200–250 mg/dL)	Assess 30‐day eKA, AKI and mortality risk post‐surgery	The SGLT2i group had a small but significantly higher risk of post‐op eKA, but lower risks of post‐op AKI and 30‐day mortality
Brekke 2025	Retro cohort	Norway	SGLT2i use	No use	No stop	Adults	T2DM	Cardiac	Elective	N/M	Assessing the link between SGLT2i use and MA	SGLT2i group had higher peri‐op ketosis and MA
Tenge 2025	Retro cohort	USA	SGLT2i use within 2 weeks before surgery	No use	N/M	Adults	N/M	Mixed (~92% non‐cardiac, ~8% cardiac)	Mixed (~86% elective, ~14% emergency)	N/M	30‐day adverse post‐op events including major complications, readmission and death	SGLT2i use was associated with a mildly lower pH but no increase in major postoperative events. Withholding pre‐op SGLT2i showed no impact on outcomes
Dixit 2025	Retro cohort	USA	SGLT2i use	No use	No stop	18 and older	T2DM	Non‐cardiac	Emergency	N/M	DKA, in the 0 to 14 days post‐op	Pre‐op SGLT2i use in emergency surgery was not linked to higher post‐op DKA risk
Auerbach 2025	Retro cohort	USA	Patients on SGLT2i stopped medications 5 days before OHS	No use	5 days before OHS	Adults	N/M	Cardiac	Elective	Glucose < 250 mg/dL + arterial pH < 7.30 + serum bicarbonate < 18 mEq/L + anion gap > 10 + positive serum or urine ketones	EKA rate	Stopping SGLT2i 5 days before OHS prevented eKA without worsening outcomes
Auerbach 2023	Retro cohort	USA	SGLT2i use	No use	No stop	18 and older	N/M	Cardiac	Elective	pH ≤ 7.32 + serum bicarbonate ≤ 18 mEq/L + blood glucose < 250 mg/dL	EKA, mortality, infection, and hospital/CVICU LOS in cardiac‐surgery patients	Post‐op eKA occurred in 15% of SGLT2i users after cardiac surgery and was linked to higher CVICU LOS
Lui 2023	Retro cohort	Hong Kong	SGLT2i prescriptions within 6 months before operations	No use	No stop	18 and older	T2DM	Mixed (~81% non‐cardiac, ~19% cardiac)	Mixed (~69% elective, ~31% emergency)	N/M	The risks of SGLT2i‐associated post‐op DKA	Pre‐op SGLT2i use increased post‐op DKA risk in T2DM patients
Iwasaki 2022	Retro cohort	Japan	SGLT2i use	No use	No stop	20 and older	T1DM or T2DM	Non‐cardiac	Elective	pH < 7.3, bicarbonate < 18 mmol/L, anion gap > 12 mmol/L, and blood glucose < 200 mg/dL	Incidence of MA with an elevated anion gap and euglycemia during the ICU stay	Use of SGLT2i is associated with a significantly higher incidence of euglycemic MA
Pitta 2025	RCT	Brazil	Empagliflozin 25 mg daily and standard care for at least 3 months	Standard‐of‐care	72 h before surgery	18 and older	T2DM	Cardiac	Elective	N/M	Post‐op AKI within 7 days of surgery	Pre‐op empagliflozin reduced AKI risk after on‐pump CABG in T2DM without compromising safety
Snel 2025	RCT	Netherlands	Empagliflozin 10 mg orally once daily, initiated 3 days pre‐op	Standard‐of‐care	Continued until 2 days post‐op	Aged between 18 and 90	DM and BMI > 25	Cardiac	Elective	Physician‐diagnosed adverse event	Between‐group difference in mean post‐op serum NGAL levels on post‐op day two	Perioperative SGLT2i significantly reduced the incidence of CSA‐AKI compared with standard care

Abbreviations: AKI, acute kidney injury; CABG, coronary artery bypass graft; CPB, cardiopulmonary bypass; Ctrl, comparator/control group; CVICU, cardiovascular intensive care unit; DM, diabetes mellitus; ED, emergency department; eKA, euglycemic ketoacidosis; LOS, length of stay; MA, metabolic acidosis; Multicenter, multiple study sites; *N*, number of patients; N/M, not mentioned; NGAL, neutrophil gelatinase‐associated lipocalin; OHS, open heart surgery; peri‐op, perioperatively; Post‐op, postoperatively; Pre‐op, preoperatively; RCT, randomised controlled trial; Retro, retrospective; SGLT2i, sodium‐glucose cotransporter‐2 inhibitor; T1DM, type 1 diabetes mellitus; T2DM, type 2 diabetes mellitus; USA, United States of America; Y, years.

**TABLE 2:** Baseline characteristics of the participants.

Study ID	Groups	Total (*N*)	Age (years, M ± SD)	Male, *n* (%)	BMI (kg/m^2^, M ± SD)	Hypertension, *n* (%)	DM *n* (%)	Obesity, *n* (%)	COPD, *n* (%)	CKD (%)	Stroke, *n* (%)	HF (%)	Cardiac surgery (%)	Non‐cardiac surgery (%)
Tallarico et al. 2025	SGLT2i use	7439	67.7 ± 8.1	7196 (96.7)	30.7 ± 6	6966 (93.6)	7004 (94.2)	N/M	1500 (20.2)	27 (0.4)	N/M	2193 (29.5)	716 (9.6)	6723 (90.4)
	No SGLT2i	33,489	67.9 ± 8.8	32,288 (96.4)	30.5 ± 6.1	31,228 (93.2)	31,316 (93.5)	N/M	6613 (19.7)	164 (0.5)	N/M	8698 (26)	3076 (9.2)	30,413 (90.8)
Brekke et al. 2025	SGLT2i use	38	66.5 ± 8.9	32 (84)	29.4 ± 6.5	31 (82)	38 (100)	N/M	N/M	N/M	N/M	N/M	38 (100)	0 (0)
	No SGLT2i	83	67.1 ± 8.2	66 (80)	28.3 ± 4.3	67 (81)	83 (100)	N/M	N/M	N/M	N/M	N/M	83 (100)	0 (0)
Tenge et al. 2025	SGLT2i use	1383	65.0 ± 12.3	867 (62.7)	29.9 ± 1.4	1007 (72.8)	1245 (90)	N/M	159 (11.5)	400 (28.9)	54 (3.9)	460 (33.3)	153 (11.1)	1230 (88.9)
	No SGLT2i	19,775	66.5 ± 14.5	10,951 (55.4)	29.1 ± 1.6	12,065 (61)	13,856 (70.1)	N/M	2446 (12.4)	8362 (42.3)	1138 (5.8)	6264 (31.7)	1594 (8.1)	18,181 (91.9)
Dixit et al. 2025	SGLT2i use	2607	59.6 ± 11.1	971 (37.2)	N/M	2453 (94.1)	2607 (100)	997 (38.2)	344 (13.2)	264 (10.1)	N/M	221 (8.5)	N/M	N/M
	No SGLT2i	32,064	64.3 ± 14.1	14,525 (45.3)	N/M	31,757 (99.1)	32,064 (100)	10,829 (33.8)	5557 (17.3)	5658 (17.6)	N/M	4389 (13.7)	N/M	N/M
Auerbach et al. 2025	SGLT2i use	48	66.7 ± 9.2	41 (85)	33.4 ± 4.7	45 (94)	36 (75)	26 (54)	1 (2.1)	N/M	N/M	N/M	48 (100)	0 (0)
	No SGLT2i	492	67 ± 10.4	310 (63)	27.8 ± 4.8	376 (78)	91 (19)	156 (32)	3 (0.6)	N/M	N/M	N/M	492 (100)	0 (0)
Auerbach et al. 2023	SGLT2i use	53	64.7 ± 9.9	39 (74)	N/M	47 (88.7)	52 (98.1)	30 (56.6)	N/M	11 (20.8%)	N/M	17 (32.1%)	53 (100%)	0 (0%)
	No SGLT2i	1601	66.3 ± 11.9	964 (60)	N/M	1268 (79.2)	411 (25.7)	437 (27.3)	N/M	222 (13.9)	N/M	302 (18.9)	1601 (100)	0 (0)
Lui et al. 2023	SGLT2i use	3419	64.1 ± 12.8	2190 (64.1)	26.8 ± 0.01	2291 (67%)	3419 (100)	N/M	N/M	N/M	458 (13.4)	545 (15.9)	N/M	N/M
	No SGLT2i	143,696	71.2 ± 16	79,450 (55.29)	25.2 ± 0	114,957 (80)	143,696 (100)	N/M	N/M	N/M	26,153 (18.2)	15,462 (10.8)	N/M	N/M
Iwasaki et al. 2022	SGLT2i use	31	68.5 ± 8.9	23 (74.2)	23.4 ± 4.2	21 (67.7)	31 (100)	N/M	N/M	3 (9.7%)	4 (12.9)	10 (32.3)	15 (48.4)	16 (51.6)
	No SGLT2i	124	71.0 ± 7.5	89 (71.8)	23.3 ± 3.9	62 (50)	120 (96.8)	N/M	N/M	10 (8.1%)	12 (9.7)	23 (18.5)	61 (49.2)	63 (50.8)
Pitta et al. 2025	SGLT2i use	71	62.1 ± 7.9	51 (71.8)	N/M	67 (94.3)	71 (100)	N/M	N/M	N/M	5 (7)	30 (42.2)	71 (100)	0 (0)
	No SGLT2i	74	61.8 ± 8.5	46 (62.1)	N/M	70 (94.5)	73 (100)	N/M	N/M	N/M	6 (8)	31 (41.9)	74 (100)	0 (0)
Snel 2025	SGLT2i use	25	69 ± 8	18 (72)	27.6 ± 3.4	13 (52)	3 (12)	N/M	N/M	CKD	N/M	N/M	25 (100)	0 (0)
	No SGLT2i	30	63 ± 10	22 (73)	28.2 ± 5.1	15 (50)	2 (7)	N/M	N/M	CKD	N/M	N/M	30 (100)	0 (0)

Abbreviations: BMI, body mass index; CKD, chronic kidney disease; COPD, chronic obstructive pulmonary disease; CVICU, cardiovascular intensive care unit; DM, diabetes mellitus; HF, heart failure; M, mean; *N*, number; N/M, not mentioned; SD, standard deviation; T2DM, type 2 diabetes mellitus; Yr, years.

### 3.3 Risk of Bias

Regarding RCTs, Pitta et al. [31] showed low risk of bias, while Snel et al. [20] showed a high risk of bias according to the ROB‐2 tool. Regarding cohort studies, six included studies had some concerns about bias [6, 19, 21, 30, 34, 35] according to the ROBINS‐I tool, while two studies showed a high risk of bias [32, 33]. Figures S1 and S2 present the authors' detailed judgements of individual domains.

### 3.4 Primary Outcomes

#### 3.4.1 Euglycemic Ketoacidosis (eKA)

**FIGURE 2:**
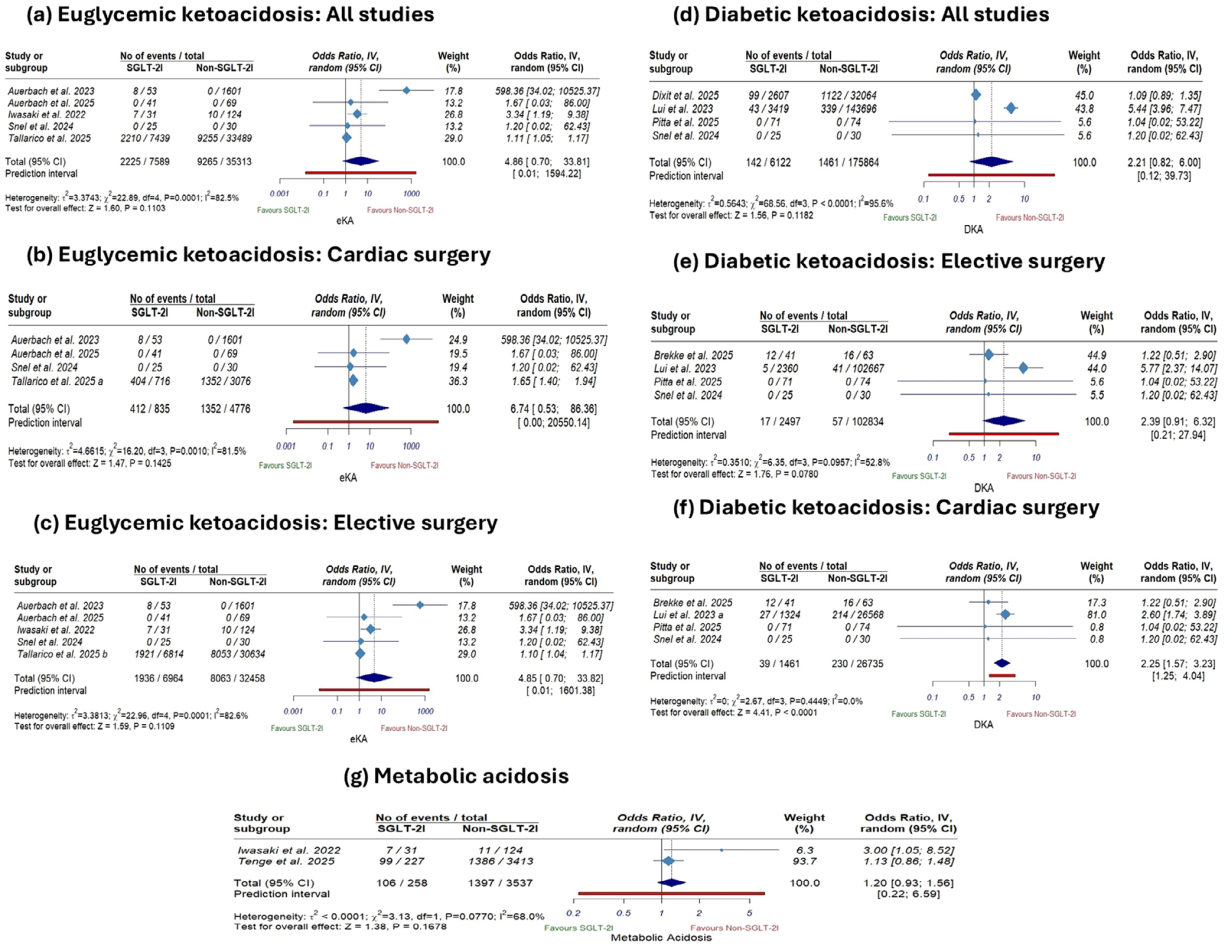
Forest plots of the primary outcomes: (a) euglycemic ketoacidosis (all studies), (b) euglycemic ketoacidosis (cardiac surgery), (c) euglycemic ketoacidosis (elective surgery), (d) diabetic ketoacidosis (all studies), (e) (elective surgery), (f) diabetic ketoacidosis (cardiac surgery), and (g) Metabolic acidosis.

The pooled analysis revealed that SGLT‐2 inhibitors use was not significantly associated with eKA in the primary analysis compared with non‐use (OR 4.86; 95% CI 0.70 to 33.81; *p* = 0.11; Figure 2a), with considerable heterogeneity (*I* ^2^ = 82.5%). This non‐significant result persisted in sensitivity analyses restricted to cardiac and elective surgeries (OR 6.74; 95% [CI 0.53 to 86.36]; *p* = 0.14; Figure 2b, OR 4.85; 95% CI [0.70 to 33.82]; *p* = 0.11; Figure 2c, respectively). However, in a leave‐one‐out analysis, we identified Auerbach et al. (2023) [32] as a primary source of heterogeneity; omitting this study showed a significantly higher eKA risk among the SGLT‐2 use group (OR 1.11; 95% CI [1.05 to 1.17]; *p* < 0.001, Figure S3a) and reduced heterogeneity (*I* ^2^ = 32%).

#### 3.4.2 Diabetic Ketoacidosis (DKA)

The primary pooled analysis revealed no statistically significant difference between the two groups (OR 2.21; 95% CI [0.82 to 6], *p* = 0.11; Figure 2d), with considerable heterogeneity (*I* ^2^ = 95.8%). This non‐significant result persisted in sensitivity analysis restricted to elective surgeries (OR 2.39; 95% CI [0.91 to 6.32], *p* = 0.07; Figure 2e); on the other hand, in sensitivity analysis restricted to cardiac surgeries, SGLT‐2 inhibitors use was associated with a significantly higher risk of DKA compared to non‐use (OR 2.25; 95% CI [1.57 to 3.23], *p* < 0.001; Figure 2f). Leave‐one‐out sensitivity analysis indicated that findings were driven by largely conflicting studies: omitting Dixit et al. (2025) [34] revealed a significant increase in DKA risk among the SGLT‐2i users group (OR 5.33; 95% CI [3.89 to 7.31]; *p* < 0.001) and resolved the heterogeneity (*I* ^2^ = 0%), whereas omitting Lui et al. (2023) [19] showed no significant difference (OR 1.09; 95% CI [0.89 to 1.35]; *p* = 0.40), and also resolved the heterogeneity (*I* ^2^ = 0%) (Figure S3b).

#### 3.4.3 Metabolic Acidosis

The pooled analysis of metabolic acidosis did not reveal a significant increase in the risk of metabolic acidosis with SGLT2i use compared to non‐use (OR 1.20, 95% CI [0.93 to 1.56]; *p* = 0.16; Figure 2g), with substantial heterogeneity (*I* ^2^ = 68%). Due to the limited number of pooled studies in this analysis, leave‐one‐out analysis was not feasible.

#### 3.4.4 Acute Kidney Injury

**FIGURE 3:**
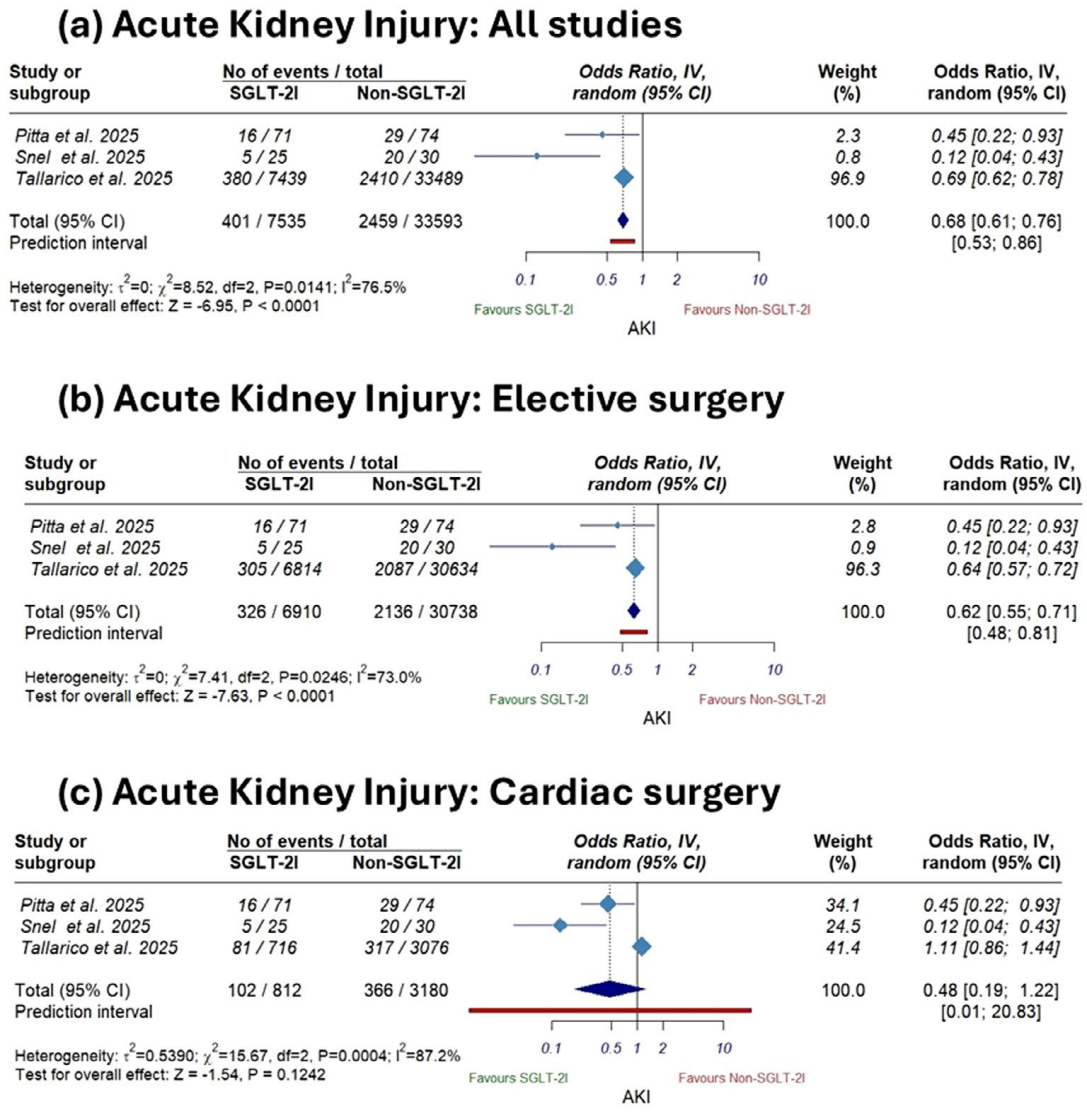
Forest plots of acute kidney injury (a) all studies, (b) elective surgery, and (c) cardiac surgery.

The primary pooled analysis revealed that SGLT‐2i use was associated with a statistically significant reduction in AKI incidence compared with non‐use (OR 0.68, 95% CI [0.61 to 0.76]; *p* < 0.0001; Figure 3a), with considerable heterogeneity (*I* ^2^ = 76.5%). This significant result persisted in sensitivity analysis restricted to elective surgeries (OR 0.62; 95% CI [0.55 to 0.71], *p* < 0.0001; Figure 3b); on the other hand, sensitivity analysis restricted to cardiac surgeries showed no significant difference between the two groups in AKI incidence (OR 0.48; 95% CI [0.19 to 1.22], *p* = 0.12; Figure 3c). Sensitivity analysis using the leave‐one‐out method demonstrated the robustness of the primary analysis, as significance was not affected by omitting any single study (Figure S3c).

### 3.5 Secondary Outcomes

#### 3.5.1 Clinical Outcomes

**FIGURE 4:**
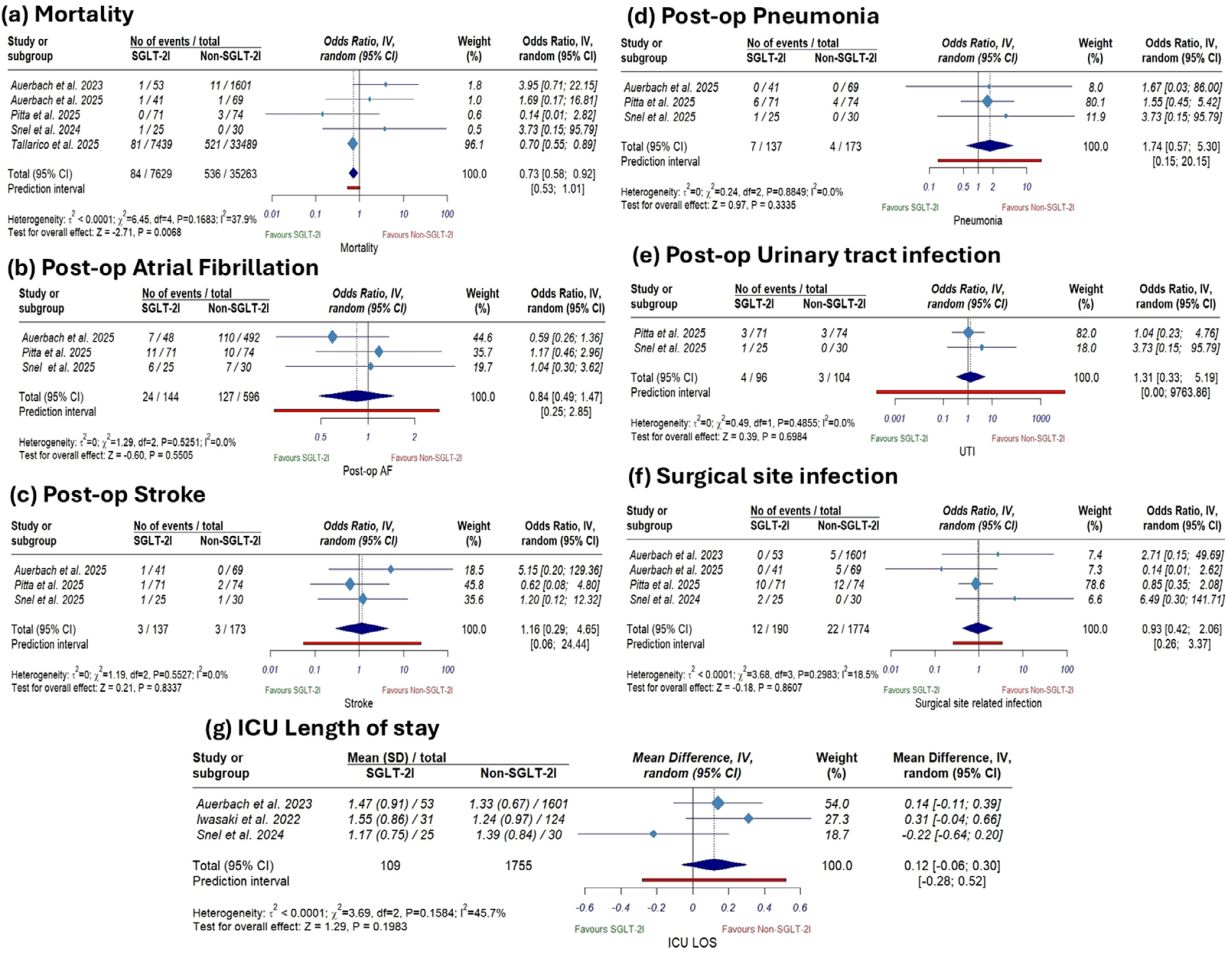
Forest plots of secondary clinical outcomes: (a) all‐cause mortality, (b) postoperative atrial fibrillation, (c) postoperative stroke, (d) postoperative pneumonia, (e) postoperative urinary tract infection, (f) surgical site infection, and (g) intensive care unit length of stay.

The pooled analysis showed that SGLT2i use was associated with significantly lower mortality rates compared to non‐SGLT2i use (OR 0.73; 95% CI [0.58 to 0.92], *p* = 0.006; Figure 4a).

This significant result persisted in sensitivity analysis restricted to elective surgeries (OR 0.77; 95% CI [0.60 to 0.99], *p* = 0.03; Figure S4a); on the other hand, sensitivity analysis restricted to cardiac surgeries showed no significant difference between the two groups in mortality (OR 1.40; 95% CI [0.68 to 2.88], *p* = 0.35; Figure S4b).

In contrast, there was no significant difference between the two groups regarding postoperative AF (OR 0.84; 95% CI [0.49 to 1.47], *p* = 0.5; Figure 4b), postoperative stroke (OR 1.16; 95% CI [0.29 to 4.65], *p* = 0.8; Figure 4c), postoperative pneumonia (OR 1.74; 95% CI [0.57 to 5.30], *p* = 0.33; Figure 4d), postoperative UTI (OR 1.31; 95% CI [0.33 to 5.19], *p* = 0.69; Figure 4e), or surgical site‐related infection (OR 0.93; 95% CI [0.42 to 2.06]; *p* = 0.86; Figure 4f). Similarly, ICU length of stay did not differ significantly between SGLT2i and non‐SGLT2i users (MD 0.12 days, 95% CI: [−0.06, 0.30]; *p* = 0.19) (Figure 4g).

The analysis revealed moderate heterogeneity for mortality (*I* ^2^ = 37.9%) and ICU length of stay (*I* ^2^ = 45.7%), minimal heterogeneity for wound infections (*I* ^2^ = 18.5%), and no heterogeneity (*I* ^2^ = 0%) for postoperative AF, stroke, pneumonia, and UTI. Leave‐one‐out analysis of mortality rates showed that omitting the Auerobach et al.(2023) study [32] resolved the heterogeneity (*I* ^2^ = 0%) without affecting the direction of the overall results or significance; however, the omission of the Tallarico et al. (2025) study [21] resulted in non‐significant results (OR 1.789; 95% CI [0.59 to 6.08], *p* = 0.28, *I* ^2^ = 20.3%; Figure S4c). On the other hand, leave‐one‐out sensitivity analyses for other secondary clinical outcomes showed robustness, as results remained consistent with the primary analysis across all omissions (Figure S5).

#### 3.5.2 Laboratory Outcomes

**FIGURE 5:**
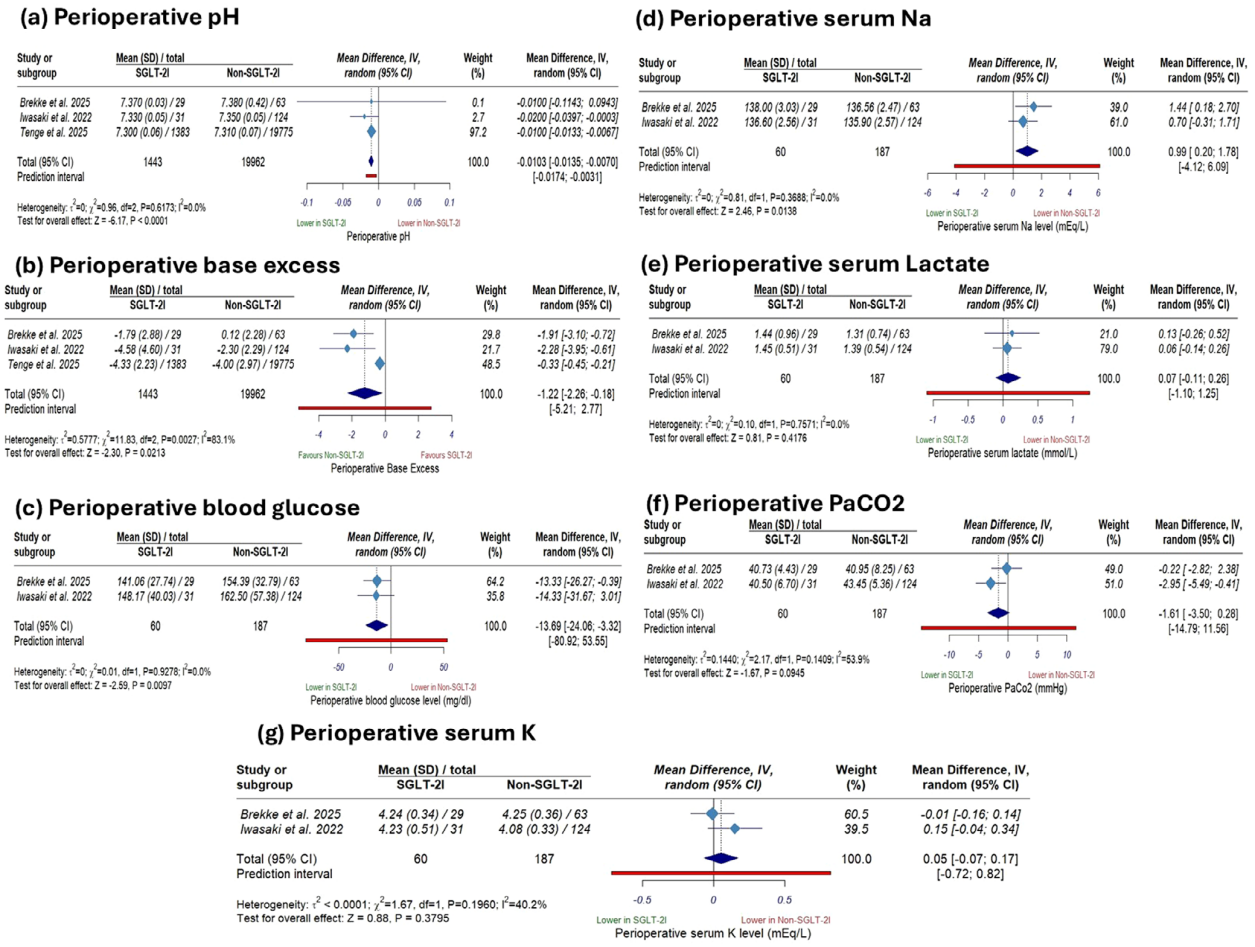
Forest plots of perioperative laboratory parameters: (a) perioperative pH, (b) perioperative base excess, (c) perioperative blood glucose, (d) perioperative serum sodium, (e) perioperative serum lactate, (f) perioperative arterial carbon dioxide tension (PaCO_2_), and (g) perioperative serum potassium.

The pooled analysis showed that compared to non‐SGLT‐2i use, SGLT‐2i use was associated with a statistically significant, but minor, reduction in perioperative pH (MD −0.0103; 95% CI [−0.0135 to −0.007], *p* < 0.0001; Figure 5a), a significant decrease in perioperative base excess (MD −1.22; 95% CI [−2.26 to −0.18], *p* = 0.02; Figure 5b), a statistically significant decline in perioperative blood glucose level (MD −13.69; 95% CI [−24.06 to −3.32], *p* = 0.009; Figure 5c), and a statistically significant but minor increase in perioperative serum Na level (MD 0.99; 95% CI [0.20 to 1.78], *p* = 0.01; Figure 5d). In contrast, there were no significant differences between the two groups in terms of perioperative lactate (MD 0.07; 95% CI [−0.11, 0.26], *p* = 0.41; Figure 5e), PaCO2 (MD −1.61; 95% CI [−3.50, 0.28], *p* = 0.09; Figure 5f), or serum K level (MD 0.05; 95% CI [−0.07, 0.17], *p* = 0.37; Figure 5g). There was considerable heterogeneity in perioperative base excess (*I* ^2^ = 83.1%), substantial heterogeneity (*I* ^2^ = 53.9%) in perioperative PaCO_2_, and moderate heterogeneity (*I* ^2^ = 40.2%) in perioperative serum K level. However, there was no heterogeneity (*I* ^2^ = 0%) in perioperative pH, perioperative lactate, perioperative blood glucose level, or perioperative serum Na level. The leave‐one‐out sensitivity analysis demonstrated the robustness of the results for perioperative pH and perioperative base excess, as significance was not affected by omitting any single study (Figure S6). Due to the limited number of pooled studies in other laboratory outcome analyses, leave‐one‐out analysis was not feasible.

## 4 Discussion

### 4.1 Executive Summary of Synthesis

The perioperative management of sodium‐glucose cotransporter‐2 (SGLT2) inhibitors is a rapidly evolving, controversial area. This systematic review and meta‐analysis, which included 246,542 patients from 10 studies published between 2022 and 2025, revealed a significant clinical dichotomy. The analysis identifies a unique metabolic liability: a propensity for ketoacidosis that is statistically masked by heterogeneity but becomes apparent when specific large‐scale observational data are included. However, the data show a compelling signal of organ protection, particularly a decrease in AKI and mortality. The unadjusted pooled risk of diabetic ketoacidosis (DKA) and euglycemic ketoacidosis (eKA) was not statistically significant (*p* = 0.11 for both comparisons). However, substantial heterogeneity (*I* ^2^ > 80%) accounts for its non‐significance. Omitting certain outliers, such as the high‐risk cohort in Auerbach et al. (2023) [32] or the emergency surgery cohort in Dixit et al. (2025) [34] in sensitivity analyses using a leave‐one‐out approach significantly shifts the statistical pattern, confirming a real metabolic risk influenced by the surgical setting.

On the other hand, the renoprotective effect is pronounced and appears independent of metabolic risk. Perioperative AKI was significantly reduced among SGLT2 inhibitor users (OR 0.68, *p* < 0.0001). The inclusion of high‐quality randomised controlled trial (RCT) data from the MERCURI‐2 study [31] provides strong evidence of efficacy in this high‐risk population, despite a non‐significant difference in cardiac surgery analyses across the broader population. Additionally, a significant mortality benefit (OR 0.73, *p* = 0.006) indicates that the pleiotropic cardiovascular advantages of SGLT2 inhibitors persist into the postoperative phase, primarily evidenced by large retrospective cohorts such as Tallarico et al. (2025) [21].

### 4.2 The Metabolic Landscape: Diabetic and Euglycemic Ketoacidosis

#### 4.2.1 Interpretation of Heterogeneity in Primary Outcomes

The primary pooled analysis of eKA and DKA showed no significant association (ORs of 4.86 and 2.21, respectively; *p* = 0.11 for both), supporting the safety of these agents. However, it would be deceptive to interpret its non‐significance superficially. The significant heterogeneity in eKA (*I* ^2^ = 82.5%) and DKA (*I* ^2^ = 95.8%) across studies, arising from diverse underlying risks or population characteristics, suggests that the average effect size may be misleading and diluted. Eliminating Auerbach et al. (2023) [32] for eKA reduced heterogeneity to 32% and resulted in a significant increase in risk (OR = 1.11, *p* < 0.001). This statistical behaviour suggests that Auerbach et al. represents an outlier with a large effect size or a discordant trend that increased the pool's variance. Auerbach et al. [32] reported a high eKA incidence of up to 15.1% in cardiac surgery, substantially greater than the typical 0.1%–0.2% seen in elective non‐cardiac populations [36]. Including a high‐magnitude outlier increased the pooled point estimate to 4.86, but the results lost significance. Omitting this outlier reveals the underlying, constant “background” risk, which shows a small but statistically significant 11% increase in the odds of eKA (OR 1.11). Similarly, eliminating Dixit et al. (2025) [34] reduced heterogeneity to 0% for DKA, revealing a high‐risk signal (OR 5.33, *p* < 0.001). The Dixit et al. study reduced the strong risk signal observed in the remaining nine studies. The challenges in discriminating between stress‐induced DKA and SGLT2i‐induced DKA, as well as the confounding effects of hyperglycemia from trauma or sepsis, may have obscured the precise effects of SGLT2 inhibitors [20, 34], because Dixit et al. investigated emergency surgery, in which metabolic stress is maximal.

#### 4.2.2 The Auerbach Anomaly: Examining the High‐Risk Signal

The risk of eDKA is a significant issue raised by Auerbach et al. (2023) regarding the perioperative use of SGLT2i in patients undergoing cardiac surgery. The environment of cardiac procedures, particularly cardiopulmonary bypass (CPB), is a key factor in the increase in eDKA risk by inducing a metabolic stress response that promotes ketogenesis [37]. Furthermore, adherence to clinical recommendations for SGLT2i management (i.e., implementation of the 3‐day drug‐withholding guidelines) during the perioperative period may have been inadequate. As a result, it's possible that patients continued SGLT2i therapy too close to surgery without sufficient washout. The study also reveals a detection bias: while such cases might go undetected in general surgical wards, greater awareness of eDKA allows for better identification in units with routine blood gas monitoring. To reduce the risks associated with SGLT2i use in cardiac surgery, increased monitoring and adherence to guidelines are necessary. The pooled eKA risk decreases from an OR of 4.86 to 1.11 when Auerbach et al.'s study is excluded, indicating that although SGLT2 inhibitors carry an inherent risk of ketosis, the surgical setting and perioperative care substantially affect the magnitude of that risk. In a highly monitored, stressful environment such as cardiac surgery, the risk is exponentially increased in the absence of adequate washout. When appropriate protocols are followed, the risk of traditional elective surgery is significantly reduced, though it remains non‐zero.

#### 4.2.3 Emergency Surgery and the Dixit Paradox

A counter‐narrative is presented in Dixit et al. (2025) [34]. Dixit et al. found no association between preoperative SGLT2i use and postoperative DKA (Average Treatment Effect, 0.2%; 95% CI, −1.7% to 2.2%) in patients undergoing emergency surgery, in which SGLT2 inhibitors cannot be withheld. Emergency surgery should provide the greatest danger mechanistically since patients have active medication on board (zero washout), are probably in a catabolic state because of acute sickness (sepsis, trauma, blockage), and have enhanced counter‐regulatory hormones. The “Dixit Paradox” could be explained by three possible explanations. The “Sick Patient” Confounder comes first: Compared with T2D patients on complex insulin regimens, those on SGLT2 inhibitors are typically healthier. Individuals with diabetes and end‐organ damage who are predisposed to DKA regardless of medication therapy may be included in the non‐SGLT2i control group, which would mask the specific drug impact in the treatment arm. The second is “Diagnostic Overshadowing”: The stress response throughout emergency surgery frequently results in hyperglycemia. In the control group, hyperglycemic DKA (classic DKA) is readily diagnosed and treated by clinicians. Patients in the SGLT2i group may experience euglycemic DKA. Cases of eDKA in the SGLT2i group may be overlooked if diagnosis is based solely on ICD‐10 codes or hyperglycemia, thereby artificially reducing the incidence rate. Finally, the “Competing Risks”: Unless beta‐hydroxybutyrate is consistently measured, which is not the norm in all centers, the high background rate of metabolic acidosis in emergency surgery (caused by lactic acidosis from hypoperfusion) may challenge the diagnosis of ketoacidosis. The statistical reality of our meta‐analysis is that Dixit et al. serves as a robust dampener regardless of these possible confounders. The remaining observational evidence warrants caution when it is eliminated: SGLT2 inhibitors increase the incidence of DKA by more than fivefold (OR 5.33). This implies that there is a real and substantial risk for the typical patient undergoing elective surgery.

#### 4.2.4 Pathophysiology of Perioperative Ketosis

Unlike classical starvation ketosis, SGLT2i‐associated ketoacidosis is a unique “fed‐state” maladaptation caused by SGLT2 block. This blockage causes obligatory glycosuria, a condition of carbohydrate depletion independent of blood glucose [38]. A high glucagon‐to‐insulin ratio results from a subsequent decrease in insulin and an increase in glucagon, due to decreased paracrine inhibition (SGLT2 receptors are expressed on alpha cells) and direct alpha‐cell stimulation [11]. This hormonal change accelerates ketone production by promoting lipolysis and activating hepatic carnitine palmitoyltransferase I (CPT‐1) [38].

Stress hormones and fasting serve as catalysts for surgery. The SGLT2 inhibitor's “open drain” on glucose prevents the hyperglycemia that normally suppresses ketogenesis, in contrast to normal physiology. As a result, the patient has euglycemic ketoacidosis, or extreme ketosis with normal blood glucose [10].

#### 4.2.5 Clinical Detection and the “Euglycemic” Trap

According to our laboratory analysis, the most significant risk factor in this illness is the “euglycemic trap.” A statistically significant decline in perioperative blood glucose was observed in our pooled analysis (MD = −13.69 mg/dL, *p* = 0.009). Although this may seem advantageous for glycemic management, it eliminates the primary warning sign, hyperglycemia, that prompts clinicians to screen for DKA.

Additionally, our pooled analysis showed that perioperative pH and base excess (MD −1.22 mEq/L) were significantly reduced [39]. According to Brekke et al. [33], a mean excess reduction of 1.22 may appear insignificant. Yet, it signifies a change at the population level; this change is characterised by a high proportion of patients (41%) exceeding the clinical acidosis threshold (BE ≤ −3). This “subclinical acidosis” suggests that many SGLT2i patients are compensated but chemically ketotic. This delicate balance can quickly be decompensated into potentially fatal acidosis by a slight extra shock, such as post‐extubation hypoventilation (respiratory acidosis) or saline‐induced hyperchloremia [39].

### 4.3 Renoprotection in the Perioperative Setting

#### 4.3.1 The AKI Reduction Signal: Statistical Versus Clinical Significance

The most significant finding of this meta‐analysis was the reduction in AKI incidence. SGLT2i users had a 32% reduction in the relative risk of AKI, according to the primary pooled analysis, which reported an odds ratio of 0.68 (*p* < 0.0001). For a perioperative intervention, this effect size is substantial and comparable to, or greater than, those of well‐studied strategies such as goal‐directed fluid therapy or remote ischemic preconditioning [40].

For elective surgery, this result is consistent across sensitivity analyses (OR = 0.62; *p* < 0.0001). It implies that the renal advantages seen in long‐term studies (DAPA‐CKD, EMPA‐KIDNEY) are [41, 42] directly applicable to the acute stress environment of surgery.

#### 4.3.2 Cardiac Surgery Specifics: The MERCURI‐2 Trial Evidence

The sensitivity analysis, which limited the analysis to cardiac surgery, revealed a non‐significant decrease in AKI (OR 0.48, *p* = 0.11) with a broad confidence interval (0.19 to 1.22) and was a source of heterogeneity in our analysis. The results of the MERCURI‐2 experiment (Snel et al., 2025) [20], a randomised controlled trial specifically designed to address this subject, stand in sharp contrast to this statistical ambiguity. Patients in the MERCURI‐2 trial were randomly assigned to receive either standard care or dapigliflozin (10 mg) before surgery. The results were precise: there was a 25% absolute risk reduction (ARR) and an AKI incidence of 28% in the dapagliflozin group compared to 53% in the standard‐of‐care group (*p* < 0.001). Furthermore, these notable decreases were observed in both AKI stages 1 and 2. These RCT results imply that the lack of significance in our pooled sensitivity analysis is likely due to a Type II error arising from heterogeneity in observational studies (e.g., small sample sizes in other cardiac cohorts or different definitions of AKI). The evidence for renoprotection in heart surgery is overwhelming when high‐quality randomised controlled trials are prioritised.

#### 4.3.3 Mechanisms of Nephroprotection

Through specific intrarenal hemodynamic mechanisms, SGLT2 inhibitors protect the kidney during surgical hypovolemia despite their diuretic effects. First, tubuloglomerular feedback (TGF) restoration: Excessive proximal sodium reabsorption in diabetes causes glomerular hyperfiltration and afferent dilatation by reducing macula densa supply. Distal salt supply is increased by SGLT2 inhibitors, which prevent this reabsorption. This restores feedback‐induced afferent vasoconstriction, normalizes glomerular pressure, and protects the glomerulus from surgical barotrauma [43, 44]. The second is the decrease in medullary hypoxia: proximal reabsorption requires substantial energy [45, 46]. SGLT2 agents significantly reduce tubular ATP consumption by blocking SGLT2 [47, 48, 49], which is essential when surgical conditions, such as CPB, restrict oxygen delivery. Snel et al. [20] observed constant postoperative hypoxia‐inducible factor‐1alpha (a hypoxia marker) levels in patients receiving SGLT2i compared to controls, confirming this better supply–demand balance. Finally, the anti‐inflammatory effects: SGLT2 inhibitors mitigate injury markers such as NGAL (Neutrophil Gelatinase‐Associated Lipocalin) and KIM‐1 (Kidney Injury Molecule‐1) [50, 51] and inhibit NLRP3 (NOD‐like receptor protein 3, LRR‐containing protein 3, and pyrin domain‐containing protein 3) inflammasome activation [47, 48]. This local anti‐inflammatory effect likely reflects a broader systemic benefit. Recent evidence also demonstrates that SGLT2 inhibitors significantly reduce inflammatory markers, such as high‐sensitivity C‐reactive protein (hsCRP), a phenomenon observed with other novel metabolic agents [49, 50].

#### 4.3.4 Reconciling the Cardiac Sensitivity Analysis

The disparity between the RCT data and the pooled observational data highlights the limits of observational research in AKI. Retrospective studies may struggle to capture the urine output criterion for AKI adequately or may conflate “structural” AKI (tubular necrosis) with “functional” AKI (transient creatinine elevation due to hemodynamic shifts). With its prospective assessment and biomarker analysis, the MERCURI‐2 [20] study offers a clearer signal. Since the drug's mode of action precisely targets the processes of harm (medullary ischemia and inflammation), we infer that the renoprotective effect is real and probably most noticeable in heart surgery.

### 4.4 Cardiovascular and Survival Outcomes

#### 4.4.1 Mortality Benefit: Assessing the Tallarico Effect

The death rate among the SGLT2i group was significantly lower in our pooled analysis (OR = 0.73, *p* = 0.006), indicating a 27% lower risk of dying following surgery. However, the strength of this finding is questionable as it became non‐significant (*p* = 0.28) when Tallarico et al. (2025) [21] was omitted in the “leave‐one‐out” analysis. Tallarico et al. conducted a large propensity‐matched analysis. This dataset dominates the meta‐analysis due to its sheer size. Despite Tallarico et al.'s careful propensity‐matching, observational studies of mortality remain vulnerable to the “Healthy User Effect.” [51]. Compared with controls, patients prescribed SGLT2 inhibitors, which are costly and require close monitoring, may have greater access to care, higher socioeconomic status, or better adherence. On the other hand, they may be more ill (recommended for CKD or heart failure). Despite accounting for comorbidities in the study, residual confounding may remain. However, given the demonstrated cardiovascular benefits in non‐surgical trials (DAPA‐HF, EMPEROR‐Reduced) [52, 53], the biological feasibility of a mortality benefit is considerable.

#### 4.4.2 Myocardial Energetics: The Thrifty Substrate Hypothesis

The “Thrifty Substrate” theory [54] may be connected to the mortality reduction in cardiac and high‐risk non‐cardiac surgery. SGLT2 inhibitors cause moderate ketosis, supplying the ischemic heart with “super‐fuel” in the form of ketone bodies such as beta‐hydroxybutyrate, which require less oxygen to produce ATP than fatty acids [55, 56, 57]. The heart's ability to use fatty acids is compromised by surgical stress, especially ischemia–reperfusion injury after CABG [58, 59]. Ketones are a highly effective energy source that supports cellular survival and cardiac contractility [60, 61]. The myocardium is probably shielded against perioperative failure, a significant cause of death in non‐cardiac surgery, by this metabolic change, as well as decreased preload and afterload.

### 4.5 Laboratory Biomarkers and Subclinical Acidosis

#### 4.5.1 The Significance of Base Excess Shifts

Clinically, the substantial decrease in base excess (MD −1.22 mEq/L) is essential. A 1.22 mEq/L decline is insignificant in healthy patients, but it indicates reduced buffering in surgical patients who are near compensation. This “base deficit” confirms that SGLT2i patients are in a unique metabolic state by indicating unmeasured anions, especially ketones, that the kidneys are unable to eliminate. Anesthesiologists must understand that a “normal” base excess in SGLT2i patients may be −2 or −3, and that any additional drop (due to hypotension or saline resuscitation, for example) can cause acidosis more quickly than in control patients [62].

#### 4.5.2 Electrolyte Derangements

The significant rise in serum sodium (MD 0.99 mEq/L) suggests that SGLT2 inhibitors have an aquaretic effect, leading to the loss of electrolyte‐free water [63]. This mild hypernatremia may cause postoperative thirst and disorientation [64]. More importantly, it indicates volume contraction, underscoring the need for cautious fluid management to balance the risk of heart failure (which necessitates restriction) with the risk of eKA (which necessitates fluid administration).

### 4.6 Regulatory Landscape and Guideline Implications

#### 4.6.1 Current Consensus Versus Emerging Evidence

According to current FDA and scientific society guidelines, SGLT2 inhibitors should be discontinued for 3 days (dapagliflozin/empagliflozin) to 4 days (ertugliflozin) before surgery. The goal of this guideline is to virtually eliminate the risk of eKA by allowing a five‐half‐life elimination period [7, 13]. Our meta‐analysis, though, identifies a necessary trade‐off. In the MERCURI‐2 study protocol, patients continued dapagliflozin until the day before surgery and then resumed it on the second postoperative day [20]. This approach significantly enhanced renal protection. Clinicians may be depriving patients of renoprotection during the most vulnerable time (the surgery itself) if they strictly follow a 4‐day washout.

#### 4.6.2 The Case for Risk‐Based Continuation

A general “stop” strategy is not ideal, as evidenced by comparisons of Dixit [34] (emergency/safe) and Snel [20] (continuation/safe) data with Auerbach [32] (cardiac/unsafe). Given the minimal risk of AKI, a 3‐day hold is advised for low‐risk elective surgery to reduce the risk of eKA. If strict ketone monitoring is possible, a 24‐h hold or continuation may be considered in high‐risk cardiac/renal situations. Through early detection and treatment, the advantages of minimising AKI (OR 0.68) and death (OR 0.73) could outweigh the risk of eKA (OR 1.11). According to Dixit's findings, do not postpone emergency surgery while closely monitoring serum ketones and the anion gap.

### 4.7 Limitations

The intrinsic issues of the included studies constrain this analysis. The severe heterogeneity in DKA analyses (*I* ^2^ = 95.8%) underscores key disparities between RCTs and observational cohorts. Retrospective coding studies likely underestimate the true incidence of eKA relative to prospective biomarker studies. Moreover, the definition of eKA varies greatly. We did not perform a certainty of evidence assessment because we included both observational studies and randomised controlled trials, which limit the applicability of a standardised grading framework such as GRADE. The mortality benefit requires validation in large‐scale randomised trials, as it is primarily based on a single major observational study. Finally, formal subgroup analysis by timing (≥ 3 days vs. < 3 days) was not feasible because there were no events (zero events) in studies with standardised withholding, thereby preventing quantitative interaction testing.

### 4.8 Future Direction and Recommendations for Future Research

Future efforts on perioperative SGLT2i management need to prioritise “vigilant use” to maximise the drug's organ‐protective advantages while mitigating metabolic concerns through fluid management and ketone monitoring protocols. Large‐scale RCTs are warranted to confirm the mortality advantage found in the Tallarico cohort. Studies should assess whether biomarker‐guided protocols, such as routine beta‐hydroxybutyrate monitoring, enable the safe use of SGLT2i in vulnerable surgical patients and whether perioperative ketosis improves cardiac survival.

## 5 Conclusion

Our meta‐analysis showed that perioperative SGLT2 inhibitor use represents a two‐edged sword. While euglycemia may mask a considerable risk of ketoacidosis, this is balanced by a significant decrease in acute renal injury and mortality. The existing practice of prolonged preoperative abstinence is questioned by the results of the MERCURI‐2 trial and our sensitivity analysis, which indicate that the benefits could outweigh the dangers for patients who are at high risk of renal injury. Therefore, minimising the withholding period as short as feasible, likely 24 h or fewer under close monitoring, may be the best approach to maintain these organ‐protective benefits.

## Author Contributions

E.B. led conceptualization, data curation, formal analysis, and methodology. A.M., N.N.E., M.B., M.S.E., A.E., and A.S. contributed to screening, extraction, and quality assessment. A.F.G. and A.M. contributed to methodology, manuscript review, writing, and formal analysis. A.I. prepared results and visualizations. A.S. assisted with manuscript review and data visualization. E.B., A.M., and A.E. contributed to writing. A.M. handled manuscript review and critical revision. A.B.‐S. contributed to supervision and manuscript editing. All authors reviewed and approved the final manuscript.

## Funding

The authors have nothing to report.

## Disclosure

*Use of Artificial Intelligence Tools*: No generative artificial intelligence or Large Language Models, including ChatGPT or related tools, were used in the preparation, writing, analysis, or editing of this manuscript.

## Ethics Statement

The authors have nothing to report.

## Consent

The authors have nothing to report.

## Conflicts of Interest

The authors declare no conflicts of interest.

## Supporting information


**Table S1:** PRISMA 2020 checklist.
**Table S2:**. Search strategy and literature search results.
**Figure S1:**. Overview of the risk of bias in the included observational studies.
**Figure S2:** Overview of the risk of bias of the included randomised clinical trials.
**Figure S3:** Leave‐one‐out sensitivity analyses for key postoperative outcomes. Panels show the pooled risk ratio with 95% confidence interval (CI) for (a) euglycemic ketoacidosis, (b) diabetic ketoacidosis, and (c) AKI.
**Figure S4:** Sensitivity analyses for mortality: (a) mortality restricted to elective surgery, (b) mortality restricted to cardiac surgery, and (c) leave‐one‐out sensitivity.
**Figure S5:** Sensitivity analyses for key secondary outcomes: (a) postoperative AF, (b) postoperative stroke, (c) postoperative pneumonia, (d) surgical site infection, and (e) surgical site infection.
**Figure S6:** Sensitivity analyses for key laboratory outcomes: (a) perioperative pH and (b) perioperative base excess.

## Data Availability

All data generated or analysed during this study are included in this published article and its Supporting Information files. Further details are available from the corresponding author upon reasonable request.

## References

[1] I. S. Padda , A. U. Mahtani , and M. Parmar , “Sodium‐Glucose Transport 2 (SGLT2) Inhibitors,” in StatPearls [Internet] [Internet] (StatPearls Publishing, 2025), https://www.ncbi.nlm.nih.gov/books/NBK576405/. 35015430

[2] M. Tanashat , A. Manasrah , and M. Abouzid , “Effects of Dapagliflozin and Empagliflozin on 6‐Min Walk Distance in Heart Failure With Preserved and Reduced Ejection Fraction: A Systematic Review and Meta‐Analysis of Randomized Controlled Trials Involving 2624 Patients,” European Journal of Clinical Pharmacology 80, no. 7 (2024): 951–963. 38498097 10.1007/s00228-024-03660-2

[3] E. Braunwald , “Gliflozins in the Management of Cardiovascular Disease,” New England Journal of Medicine 386, no. 21 (2022): 2024–2034. 35613023 10.1056/NEJMra2115011

[4] D. K. McGuire , W. J. Shih , F. Cosentino , et al., “Association of SGLT2 Inhibitors With Cardiovascular and Kidney Outcomes in Patients With Type 2 Diabetes: A Meta‐Analysis,” JAMA Cardiology 6, no. 2 (2021): 148–158. 33031522 10.1001/jamacardio.2020.4511 PMC7542529

[5] G. Y. Eljadid , M. S. Rakab , A. Mansour , et al., “Empagliflozin Effect on Left Cardiac Parameters in Acute Coronary Syndrome: A Systematic Review and Meta‐Analysis of Randomized Controlled Trials,” Cureus [Internet] 16, no. 9 (2024): 1–12, https://cureus.com/articles/275486‐empagliflozin‐effect‐on‐left‐cardiac‐parameters‐in‐acute‐coronary‐syndrome‐a‐systematic‐review‐and‐meta‐analysis‐of‐randomized‐controlled‐trials. 10.7759/cureus.69229 PMC11470159 39398777

[6] Y. Iwasaki , Y. Sasabuchi , S. Horikita , et al., “The Effect of Preoperative Sodium‐Glucose Cotransporter 2 Inhibitors on the Incidence of Perioperative Metabolic Acidosis: A Retrospective Cohort Study,” BMC Endocrine Disorders 22, no. 1 (2022): 209. 35987618 10.1186/s12902-022-01126-z PMC9392326

[7] “FDA Revises Labels of SGLT2 Inhibitors for Diabetes to Include Warnings About Too Much Acid in the Blood and Serious Urinary Tract Infections | FDA [Internet],” (2025), https://www.fda.gov/drugs/drug‐safety‐and‐availability/fda‐revises‐labels‐sglt2‐inhibitors‐diabetes‐include‐warnings‐about‐too‐much‐acid‐blood‐and‐serious.

[8] A. Koceva and N. A. Kravos Tramšek , “From Sweet to Sour: SGLT‐2‐Inhibitor‐Induced Euglycemic Diabetic Ketoacidosis,” Journal of Personalized Medicine 14, no. 7 (2024): 665. 39063919 10.3390/jpm14070665 PMC11277626

[9] B. Garcia , M. Ostermann , and M. Legrand , “How to Manage Sodium‐Glucose Cotransporter‐2 Inhibitors in the Critically Ill Patient?,” Intensive Care Medicine 51, no. 1 (2025): 143–145. 39621048 10.1007/s00134-024-07704-0

[10] A. L. Peters , E. O. Buschur , J. B. Buse , P. Cohan , J. C. Diner , and I. B. Hirsch , “Euglycemic Diabetic Ketoacidosis: A Potential Complication of Treatment With Sodium–Glucose Cotransporter 2 Inhibition,” Diabetes Care 38, no. 9 (2015): 1687–1693. 26078479 10.2337/dc15-0843 PMC4542270

[11] C. Bonner , J. Kerr‐Conte , V. Gmyr , et al., “Inhibition of the Glucose Transporter SGLT2 With Dapagliflozin in Pancreatic Alpha Cells Triggers Glucagon Secretion,” Nature Medicine 21, no. 5 (2015): 512–517. 10.1038/nm.3828 25894829

[12] E. Balbaa , A. A. Ibrahim , M. Bazzazeh , et al., “Non‐Fasting Versus Fasting Before Percutaneous Cardiac Procedures: A Systematic Review and Meta‐Analysis of Randomized Controlled Trials,” Perioperative Medicine (London, England) 14, no. 1 (2025): 24. 40022233 10.1186/s13741-024-00485-6 PMC11869692

[13] S. M. Hwang , A. S. Abcejo , A. K. Jacob , J. M. Raiten , and M. S. Mundi , “Editorial: Euglycemic Ketoacidosis Concerns in Perioperative Use of SGLT2 Inhibitors: Re‐Examining Current Recommendations,” APSF Newsletter 40, no. 1 (2025): 13–15, https://www.google.com/search?q=Hwang+SM%2C+Abcejo+AS%2C+Jacob+AK%2C+Raiten+JM%2C+Mundi+MS.+Editorial%3A+Euglycemic+ketoacidosis+concerns+in+perioperative+use+of+SGLT2+inhibitors%3A+Re‐examining+current+recommendations.+APSF+Newsletter.+2025+Feb%3B40(1)%3A13%E2%80%9315.+Available+from%3A+https%3A%2F%2Fwww.apsf.org.&oq=Hwang+SM%2C+Abcejo+AS%2C+Jacob+AK%2C+Raiten+JM%2C+Mundi+MS.+Editorial%3A+Euglycemic+ketoacidosis+concerns+in+perioperative+use+of+SGLT2+inhibitors%3A+Re‐examining+current+recommendations.+APSF+Newsletter.+2025+Feb%3B40(1)%3A13%E2%80%9315.+Available+from%3A+https%3A%2F%2Fwww.apsf.org.+&gs_lcrp=EgZjaHJvbWUyBggAEEUYOTIHCAEQIRiPAjIHCAIQIRiPAtIBCTEwNTRqMGoxNagCCLACAfEFQvhwTWSehC8&sourceid=chrome&ie=UTF‐8.

[14] D. Juneja , P. Nasa , R. Jain , and O. Singh , “Sodium‐Glucose Cotransporter‐2 Inhibitors Induced Euglycemic Diabetic Ketoacidosis: A Meta Summary of Case Reports,” World Journal of Diabetes 14, no. 8 (2023): 1314–1322. 37664476 10.4239/wjd.v14.i8.1314 PMC10473945

[15] E. Sampani , P. Sarafidis , C. Dimitriadis , et al., “Severe Euglycemic Diabetic Ketoacidosis of Multifactorial Etiology in a Type 2 Diabetic Patient Treated With Empagliflozin: Case Report and Literature Review,” BMC Nephrology 21, no. 1 (2020): 276. 32669085 10.1186/s12882-020-01930-6 PMC7364613

[16] P. B. Mehta , A. Robinson , D. Burkhardt , and R. J. Rushakoff , “Inpatient Perioperative Euglycemic Diabetic Ketoacidosis due to Sodium‐Glucose Cotransporter‐2 Inhibitors ‐ Lessons From a Case Series and Strategies to Decrease Incidence,” Endocrine Practice 28, no. 9 (2022): 884–888. 35753675 10.1016/j.eprac.2022.06.006

[17] B. Chacko , M. Whitley , U. Beckmann , K. Murray , and M. Rowley , “Postoperative Euglycaemic Diabetic Ketoacidosis Associated With Sodium‐Glucose Cotransporter‐2 Inhibitors (Gliflozins): A Report of Two Cases and Review of the Literature,” Anaesthesia and Intensive Care 46, no. 2 (2018): 215–219. 29519226 10.1177/0310057X1804600212

[18] A. Lau , S. Bruce , E. Wang , R. Ree , K. Rondi , and A. Chau , “Perioperative Implications of Sodium‐Glucose Cotransporter‐2 Inhibitors: A Case Series of Euglycemic Diabetic Ketoacidosis in Three Patients After Cardiac Surgery,” Canadian Journal of Anesthesia=Journal Canadien D'anesthésie 65, no. 2 (2018): 188–193. 10.1007/s12630-017-1018-6 29168157

[19] D. T. W. Lui , T. Wu , I. C. H. Au , et al., “A Population‐Based Study of SGLT2 Inhibitor‐Associated Postoperative Diabetic Ketoacidosis in Patients With Type 2 Diabetes,” Drug Safety 46, no. 1 (2023): 53–64. 36289137 10.1007/s40264-022-01247-3

[20] L. I. P. Snel , M. J. P. Oosterom‐Eijmael , E. Rampanelli , et al., “The Effects of Sodium‐Glucose Transporter 2 Inhibition on Cardiac Surgery‐Associated Acute Kidney Injury: An Open‐Label Randomized Pilot Study,” Journal of Clinical Anesthesia 103 (2025): 111811. 40153894 10.1016/j.jclinane.2025.111811

[21] “Postoperative Outcomes Among Sodium‐Glucose Cotransporter 2 Inhibitor Users—PubMed [Internet],” (2025), https://pubmed.ncbi.nlm.nih.gov/40305034/. 10.1001/jamasurg.2025.0940 PMC12044541 40305034

[22] M. Cumpston , T. Li , M. J. Page , et al., “Updated Guidance for Trusted Systematic Reviews: A New Edition of the Cochrane Handbook for Systematic Reviews of Interventions,” Cochrane Database of Systematic Reviews 10, no. 10 (2019): ED000142. 31643080 10.1002/14651858.ED000142 PMC10284251

[23] “The PRISMA 2020 Statement: An Updated Guideline for Reporting Systematic Reviews | the BMJ [Internet],” (2025), https://www.bmj.com/content/372/bmj.n71. 10.1136/bmj.n71 PMC8005924 33782057

[24] M. Ouzzani , H. Hammady , Z. Fedorowicz , and A. Elmagarmid , “Rayyan—A Web and Mobile App for Systematic Reviews,” Systematic Reviews 5, no. 1 (2016): 210. 27919275 10.1186/s13643-016-0384-4 PMC5139140

[25] J. A. Sterne , J. Savović , M. J. Page , et al., “RoB 2: A Revised Tool for Assessing Risk of Bias in Randomised Trials,” BMJ [Internet] 366 (2025): l4898, https://www.bmj.com/content/366/bmj.l4898. 10.1136/bmj.l4898 31462531

[26] J. A. Sterne , M. A. Hernán , B. C. Reeves , et al., “ROBINS‐I: A Tool for Assessing Risk of Bias in Non‐Randomised Studies of Interventions,” BMJ 355 (2016): i4919. 27733354 10.1136/bmj.i4919 PMC5062054

[27] F. Naeem , S. Tabassum , M. Burhan , et al., “Efficacy and Safety of Colchicine Post Myocardial Infarction: A Systematic Review, Meta‐Analysis and Meta‐Regression Analysis of Randomized Clinical Trials,” European Journal of Clinical Pharmacology 81, no. 9 (2025): 1257–1274. 40548988 10.1007/s00228-025-03869-9

[28] E. Balbaa , A. F. Gadelmawla , A. M. Tawfik , et al., “Protamine Sulphate for Heparin Reversal in Percutaneous Cardiac Interventions: A Systematic Review and Meta‐Analysis of Randomized Controlled Trials,” Naunyn‐Schmiedeberg's Archives of Pharmacology 398 (2025): 16507–16520. 40613937 10.1007/s00210-025-04369-4 PMC12678601

[29] M. Egger , G. Davey Smith , M. Schneider , and C. Minder , “Bias in Meta‐Analysis Detected by a Simple, Graphical Test,” BMJ 315, no. 7109 (1997): 629–634. 9310563 10.1136/bmj.315.7109.629 PMC2127453

[30] J. S. Auerbach , A. Alnajar , S. S. Patel , et al., “Retrospective Chart Review of Euglycemic Diabetic Ketoacidosis Rates and Outcomes Postimplementation of Sodium Glucose Cotransporter 2 Inhibitor Use Stoppage 5 Days Before Open Heart Surgery,” Journal of Cardiothoracic and Vascular Anesthesia [Internet] 39 (2025): 1441–1450, https://www.jcvaonline.com/article/S1053‐0770(25)00170‐3/fulltext. 40090790 10.1053/j.jvca.2025.02.030

[31] F. G. Pitta , E. G. Lima , C. A. M. Tavares , et al., “Empagliflozin in Patients With Type 2 Diabetes Undergoing On‐Pump CABG: The POST‐CABGDM Randomized Clinical Trial,” Diabetes Care 48, no. 6 (2025): 988–995. 40233024 10.2337/dc24-2807

[32] “Postcardiac Surgery Euglycemic Diabetic Ketoacidosis in Patients on Sodium‐Glucose Cotransporter 2 Inhibitors—PubMed [Internet],” (2025), https://pubmed.ncbi.nlm.nih.gov/36872114/. 10.1053/j.jvca.2023.01.041 36872114

[33] H. K. Brekke , G. Holmaas , M. C. Astor , et al., “Metabolic Acidosis in Patients With Diabetes 2 Undergoing Cardiac Surgery: The Impact of SGLT2 Inhibitor Use: A Retrospective Cohort Study,” European Journal of Anaesthesiology 42, no. 2 (2025): 152–161. 39450428 10.1097/EJA.0000000000002090

[34] A. A. Dixit , B. T. Bateman , M. T. Hawn , M. C. Odden , and E. C. Sun , “Preoperative SGLT2 Inhibitor Use and Postoperative Diabetic Ketoacidosis,” JAMA Surgery 160, no. 4 (2025): 423–430. 39969891 10.1001/jamasurg.2024.7082 PMC11840685

[35] “Preoperative SGLT‐2 Inhibitor Use and Risk of Adverse Postoperative Events: A Single‐Centre Retrospective Observational Study—PubMed [Internet],” (2025), https://pubmed.ncbi.nlm.nih.gov/39863469/. 10.1016/j.bja.2024.12.024 PMC11947582 39863469

[36] P. B. Mehta , A. Robinson , D. Burkhardt , and R. J. Rushakoff , “Inpatient Perioperative Euglycemic Diabetic Ketoacidosis due to Sodium‐Glucose Cotransporter‐2 Inhibitors ‐ Lessons From a Case Series and Strategies to Decrease Incidence,” Endocrine Practice 28, no. 9 (2022): 884–888. 35753675 10.1016/j.eprac.2022.06.006

[37] A. Branco , R. Fatima , K. Liblik , R. Jackson , D. Payne , and M. El‐Diasty , “Euglycemic Diabetic Ketoacidosis Associated With Sodium‐Glucose Cotransporter‐2 Inhibitors After Cardiac Surgery: A Review of Current Literature,” Journal of Cardiothoracic and Vascular Anesthesia 36, no. 10 (2022): 3877–3886. 35863986 10.1053/j.jvca.2022.06.008

[38] E. Ferrannini , E. Muscelli , S. Frascerra , et al., “Metabolic Response to Sodium‐Glucose Cotransporter 2 Inhibition in Type 2 Diabetic Patients,” Journal of Clinical Investigation 124, no. 2 (2014): 499–508. 24463454 10.1172/JCI72227 PMC3904627

[39] D. A. Milder , T. Y. Milder , and P. C. A. Kam , “Sodium‐Glucose Co‐Transporter Type‐2 Inhibitors: Pharmacology and Peri‐Operative Considerations,” Anaesthesia 73, no. 8 (2018): 1008–1018. 29529345 10.1111/anae.14251

[40] C. C. Zhao , Y. Ye , Z. Q. Li , X. H. Wu , C. Zhao , and Z. J. Hu , “Effect of Goal‐Directed Fluid Therapy on Renal Function in Critically Ill Patients: A Systematic Review and Meta‐Analysis,” Renal Failure 44, no. 1 (2022): 777–789. 35535511 10.1080/0886022X.2022.2072338 PMC9103701

[41] “Empagliflozin in Patients With Chronic Kidney Disease | New England Journal of Medicine [Internet],” (2026), https://www.nejm.org/doi/full/10.1056/NEJMoa2204233.

[42] H. J. L. Heerspink , B. V. Stefánsson , R. Correa‐Rotter , et al., “Dapagliflozin in Patients With Chronic Kidney Disease,” New England Journal of Medicine 383, no. 15 (2020): 1436–1446. 32970396 10.1056/NEJMoa2024816

[43] B. R. Góes‐Santos , P. C. Castro , A. C. C. Girardi , L. M. Antunes‐Correa , and A. P. Davel , “Vascular Effects of SGLT2 Inhibitors: Evidence and Mechanisms,” American Journal of Physiology. Cell Physiology 329, no. 4 (2025): C1150–C1160. 40908107 10.1152/ajpcell.00569.2025

[44] “The Proximal Tubule in the Pathophysiology of the Diabetic Kidney | American Journal of Physiology‐Regulatory, Integrative and Comparative Physiology | American Physiological Society [Internet],” (2026), https://journals.physiology.org/doi/full/10.1152/ajpregu.00809.2010. 10.1152/ajpregu.00809.2010 PMC3094037 21228342

[45] M. A. A. Fuchs and M. Wolf , “Renal Proximal Tubule Cells: Power and Finesse,” Journal of Clinical Investigation 133, no. 9 (2023): e169607. 37115697 10.1172/JCI169607 PMC10145182

[46] G. S. Papaetis , “SGLT2 Inhibitors, Intrarenal Hypoxia and the Diabetic Kidney: Insights Into Pathophysiological Concepts and Current Evidence,” Archives of Medical Sciences. Atherosclerotic Diseases 8 (2023): e155–e168. 38283924 10.5114/amsad/176658 PMC10811536

[47] Q. Ke , C. Shi , Y. Lv , et al., “SGLT2 Inhibitor Counteracts NLRP3 Inflammasome via Tubular Metabolite Itaconate in Fibrosis Kidney,” FASEB Journal 36, no. 1 (2022): e22078. 34918381 10.1096/fj.202100909RR

[48] S. R. Kim , S. G. Lee , S. H. Kim , et al., “SGLT2 Inhibition Modulates NLRP3 Inflammasome Activity via Ketones and Insulin in Diabetes With Cardiovascular Disease,” Nature Communications 11, no. 1 (2020): 2127. 10.1038/s41467-020-15983-6 PMC7195385 32358544

[49] “Anti‐Inflammatory Effects of Empagliflozin in Patients With Type 2 Diabetes and Insulin Resistance | Diabetology & Metabolic Syndrome [Internet],” 2026, https://link.springer.com/article/10.1186/s13098‐018‐0395‐5. 10.1186/s13098-018-0395-5 PMC6299593 30574207

[50] “Safety and Efficacy of Glucagon‐Like Peptide‐1 Receptor Agonists in Patients With Obstructive Sleep Apnea: A Systematic Review and Meta‐Analysis of Randomized Controlled Trials—PubMed [Internet],” (2026), https://pubmed.ncbi.nlm.nih.gov/40144943/. 10.1080/20018525.2025.2484048 PMC11938315 40144943

[51] W. H. Shrank , A. R. Patrick , and M. Alan Brookhart , “Healthy User and Related Biases in Observational Studies of Preventive Interventions: A Primer for Physicians,” Journal of General Internal Medicine 26, no. 5 (2011): 546–550. 21203857 10.1007/s11606-010-1609-1 PMC3077477

[52] M. Packer , S. D. Anker , J. Butler , et al., “Cardiovascular and Renal Outcomes With Empagliflozin in Heart Failure,” New England Journal of Medicine [Internet] 383 (2026): 1413–1424, https://www.nejm.org/doi/full/10.1056/NEJMoa2022190. 10.1056/NEJMoa2022190 32865377

[53] M. M. JJ , S. D. Solomon , S. E. Inzucchi , et al., “Dapagliflozin in Patients With Heart Failure and Reduced Ejection Fraction,” New England Journal of Medicine [Internet] 381 (2026): 1995–2008, https://www.nejm.org/doi/full/10.1056/NEJMoa1911303. 10.1056/NEJMoa1911303 31535829

[54] “CV Protection in the EMPA‐REG OUTCOME Trial: A “Thrifty Substrate” Hypothesis—PubMed [Internet],” (2026), https://pubmed.ncbi.nlm.nih.gov/27289126/. 10.2337/dc16-0330 27289126

[55] M. Koutentakis , J. Kuciński , D. Świeczkowski , S. Surma , K. J. Filipiak , and A. Gąsecka , “The Ketogenic Effect of SGLT‐2 Inhibitors‐Beneficial or Harmful?,” Journal of Cardiovascular Development and Disease 10, no. 11 (2023): 465. 37998523 10.3390/jcdd10110465 PMC10672595

[56] S. Mudaliar , S. Alloju , and R. R. Henry , “Can a Shift in Fuel Energetics Explain the Beneficial Cardiorenal Outcomes in the EMPA‐REG OUTCOME Study? A Unifying Hypothesis,” Diabetes Care 39, no. 7 (2016): 1115–1122. 27289124 10.2337/dc16-0542

[57] G. D. Lopaschuk and S. Verma , “Empagliflozin's Fuel Hypothesis: Not So Soon,” Cell Metabolism 24, no. 2 (2016): 200–202. 27508868 10.1016/j.cmet.2016.07.018

[58] G. D. Lopaschuk , J. R. Ussher , C. D. L. Folmes , J. S. Jaswal , and W. C. Stanley , “Myocardial Fatty Acid Metabolism in Health and Disease,” Physiological Reviews 90, no. 1 (2010): 207–258. 20086077 10.1152/physrev.00015.2009

[59] D. M. Yellon and D. J. Hausenloy , “Myocardial Reperfusion Injury,” New England Journal of Medicine 357, no. 11 (2007): 1121–1135. 17855673 10.1056/NEJMra071667

[60] “Multi‐Dimensional Roles of Ketone Bodies in Fuel Metabolism, Signaling, and Therapeutics—PubMed [Internet],” (2026), https://pubmed.ncbi.nlm.nih.gov/28178565/. 10.1016/j.cmet.2016.12.022 PMC5313038 28178565

[61] R. Nielsen , N. Møller , L. C. Gormsen , et al., “Cardiovascular Effects of Treatment With the Ketone Body 3‐Hydroxybutyrate in Chronic Heart Failure Patients,” Circulation 139, no. 18 (2019): 2129–2141. 30884964 10.1161/CIRCULATIONAHA.118.036459 PMC6493702

[62] R. M. Goldenberg , L. D. Berard , A. Y. Y. Cheng , et al., “SGLT2 Inhibitor‐Associated Diabetic Ketoacidosis: Clinical Review and Recommendations for Prevention and Diagnosis,” Clinical Therapeutics 38, no. 12 (2016): 2654–2664.e1. 28003053 10.1016/j.clinthera.2016.11.002

[63] N. A. Mordi , I. R. Mordi , J. S. Singh , R. J. McCrimmon , A. D. Struthers , and C. C. Lang , “Renal and Cardiovascular Effects of SGLT2 Inhibition in Combination With Loop Diuretics in Patients With Type 2 Diabetes and Chronic Heart Failure,” Circulation 142, no. 18 (2020): 1713–1724. 32865004 10.1161/CIRCULATIONAHA.120.048739 PMC7594536

[64] “Frontiers | Association Between Hypernatremia and Delirium After Cardiac Surgery: A Nested Case‐Control Study [Internet],” (2026), https://www.frontiersin.org/journals/cardiovascular‐medicine/articles/10.3389/fcvm.2022.828015/full. 10.3389/fcvm.2022.828015 PMC8959150 35355967

