# Supplementary material for: Renal Protection at a Metabolic Cost: A Systematic Review and Meta‐Analysis of Perioperative Use of Sodium–Glucose Cotransporter 2 Inhibitors
Source: Endocrinol Diabetes Metab. 2026 Feb 21;9(2):e70180. doi: 10.1002/edm2.70180 (PMC12928041; doi:10.1002/edm2.70180)
Supplement: Supplementary file 1 — Table S1: PRISMA 2020 checklist. Table S2:. Search strategy and literature search results. Figure S1:. Overview of the risk of bias in the included observational studies. Figure S2: Overview of the risk of bias of the included randomised clinical trials. Figure S3: Leave‐one‐out sensitivity analyses for key postoperative outcomes. Panels show the pooled risk ratio with 95% confidence interval (CI) for (a) euglycemic ketoacidosis, (b) diabetic ketoacidosis, and (c) AKI. Figure S4: Sensitivity analyses for mortality: (a) mortality restricted to elective surgery, (b) mortality restricted to cardiac surgery, and (c) leave‐one‐out sensitivity. Figure S5: Sensitivity analyses for key secondary outcomes: (a) postoperative AF, (b) postoperative stroke, (c) postoperative pneumonia, (d) surgical site infection, and (e) surgical site infection. Figure S6: Sensitivity analyses for key laboratory outcomes: (a) perioperative pH and (b) perioperative base excess. [file EDM2-9-e70180-s001.docx]

**Supplementary Materials**

**Renoprotection at a Metabolic Cost: A Systematic Review and Meta-Analysis of Perioperative Sodium-Glucose Cotransporter-2 Inhibitors Use.**

The authors have provided this supplementary material to demonstrate additional information about this study.

**Index.**

| **Supplementary Content:** | | |
| --- | --- | --- |
| 1. **Tables:** | | **Page** |
| **1** | **Table S1.** PRISMA 2020 checklist. | 3 |
| **2** | **Table S2.** Search strategy and literature search results. | 6 |
| **b. Figures:** | | **Page** |
| **1** | **Figure S1.** Overview of the risk of bias in the included observational studies. | 7 |
| **2** | **Figure S2.** Overview of the risk of bias of the included randomized clinical trials. | 8 |
| **3** | **Figure S3.** Leave-one-out sensitivity analyses for key postoperative outcomes. Panels show the pooled risk ratio with 95% confidence interval (CI) for (a) euglycemic ketoacidosis, (b) diabetic ketoacidosis, and (c) AKI. | 9 |
| **4** | **Figure S4.** Sensitivity analyses for mortality: (a) mortality restricted to elective surgery, (b) mortality restricted to cardiac surgery, and (c) leave-one-out sensitivity. | 10 |
| **5** | **Figure S5.** Sensitivity analyses for key secondary outcomes: (a) postoperative AF, (b) postoperative stroke, (c) postoperative pneumonia, (d) surgical site infection, and (e) surgical site infection. | 11 |
| **6** | **Figure S6.** Sensitivity analyses for key laboratory outcomes: (a) perioperative pH and (b) perioperative base excess. | 12 |

**Table S1:** PRISMA 2020 checklist.

| **Section and Topic** | **Item #** | **Checklist item** | **Location where item is reported** |
| --- | --- | --- | --- |
| **TITLE** | | | |
| Title | 1 | Identify the report as a systematic review. | Title page |
| **ABSTRACT** | | | |
| Abstract | 2 | See the PRISMA 2020 for Abstracts checklist. | Page 3,4 |
| **INTRODUCTION** | | | |
| Rationale | 3 | Describe the rationale for the review in the context of existing knowledge. | Page 5,6 |
| Objectives | 4 | Provide an explicit statement of the objective(s) or question(s) the review addresses. | Page 5,6 |
| **METHODS** | | | |
| Eligibility criteria | 5 | Specify the inclusion and exclusion criteria for the review and how studies were grouped for the syntheses. | Page 7.8, subsection 2.3 |
| Information sources | 6 | Specify all databases, registers, websites, organisations, reference lists and other sources searched or consulted to identify studies. Specify the date when each source was last searched or consulted. | Page 7, subsection 2.2 |
| Search strategy | 7 | Present the full search strategies for all databases, registers, and websites, including any filters and limits used. | Page 7, subsection 2.2 Sup material, table S2 |
| Selection process | 8 | Specify the methods used to decide whether a study met the inclusion criteria of the review, including how many reviewers screened each record and each report retrieved, whether they worked independently, and if applicable, details of automation tools used in the process. | Page 8, subsection 2.4 |
| Data collection process | 9 | Specify the methods used to collect data from reports, including how many reviewers collected data from each report, whether they worked independently, any processes for obtaining or confirming data from study investigators, and if applicable, details of automation tools used in the process. | Page 8,9, subsection 2.5 |
| Data items | 10a | List and define all outcomes for which data were sought. Specify whether all results that were compatible with each outcome domain in each study were sought (e.g. for all measures, time points, analyses), and if not, the methods used to decide which results to collect. | Page 8,9, subsection 2.5 |
|  | 10b | List and define all other variables for which data were sought (e.g. participant and intervention characteristics, funding sources). Describe any assumptions made about any missing or unclear information. | Page 8,9, subsection 2.5 |
| Study risk of bias assessment | 11 | Specify the methods used to assess risk of bias in the included studies, including details of the tool(s) used, how many reviewers assessed each study and whether they worked independently, and if applicable, details of automation tools used in the process. | Page 9, subsection 2.6 |
| Effect measures | 12 | Specify for each outcome the effect measure(s) (e.g. risk ratio, mean difference) used in the synthesis or presentation of results. | Page 9, subsection 2.7 |
| Synthesis methods | 13a | Describe the processes used to decide which studies were eligible for each synthesis (e.g. tabulating the study intervention characteristics and comparing against the planned groups for each synthesis (item #5)). | Page 9, subsection 2.7 |
|  | 13b | Describe any methods required to prepare the data for presentation or synthesis, such as handling of missing summary statistics, or data conversions. | Page 9, subsection 2.7 |
|  | 13c | Describe any methods used to tabulate or visually display results of individual studies and syntheses. | Page 9, subsection 2.7 |
|  | 13d | Describe any methods used to synthesize results and provide a rationale for the choice(s). If meta-analysis was performed, describe the model(s), method(s) to identify the presence and extent of statistical heterogeneity, and software package(s) used. | Page 9, subsection 2.7 |
|  | 13e | Describe any methods used to explore plausible causes of heterogeneity among study results (e.g. subgroup analysis, meta-regression). | Page 10, subsection 2.7 |
|  | 13f | Describe any sensitivity analyses conducted to assess robustness of the synthesized results. | Page 9, subsection 2.7 |
| Reporting bias assessment | 14 | Describe any methods used to assess risk of bias due to missing results in a synthesis (arising from reporting biases). | Not applicable |
| Certainty assessment | 15 | Describe any methods used to assess certainty (or confidence) in the body of evidence for an outcome. | Not applicable |
| **RESULTS** | | | |
| Study selection | 16a | Describe the results of the search and selection process, from the number of records identified in the search to the number of studies included in the review, ideally using a flow diagram. | Page 11, subsection 3.1 |
|  | 16b | Cite studies that might appear to meet the inclusion criteria, but which were excluded, and explain why they were excluded. | Page 11, subsection 3.1 |
| Study characteristics | 17 | Cite each included study and present its characteristics. | Table 1 & 2, Page 11, subsection 3.2 |
| Risk of bias in studies | 18 | Present assessments of risk of bias for each included study. | Page 12, subsection 3.3 |
| Results of individual studies | 19 | For all outcomes, present, for each study: (a) summary statistics for each group (where appropriate) and (b) an effect estimates and its precision (e.g. confidence/credible interval), ideally using structured tables or plots. | Table 1 & 2, Page 11, subsection 3.2 |
| Results of syntheses | 20a | For each synthesis, briefly summarise the characteristics and risk of bias among contributing studies. | Table 1 & 2, Page 11, subsection 3.2 |
|  | 20b | Present results of all statistical syntheses conducted. If meta-analysis was done, present for each the summary estimate and its precision (e.g. confidence/credible interval) and measures of statistical heterogeneity. If comparing groups, describe the direction of the effect. | Pages 12-14, subsections 3.4-3.5 |
|  | 20c | Present results of all investigations of plausible causes of heterogeneity among study results. | Page 11, subsection 3.2 |
|  | 20d | Present results of all sensitivity analyses conducted to assess the robustness of the synthesized results. | Pages 12-14, subsections 3.4-3.5 |
| Reporting biases | 21 | Present assessments of risk of bias due to missing results (arising from reporting biases) for each synthesis assessed. | Not applicable |
| Certainty of evidence | 22 | Present assessments of certainty (or confidence) in the body of evidence for each outcome assessed. | Not applicable |
| **DISCUSSION** | | | |
| Discussion | 23a | Provide a general interpretation of the results in the context of other evidence. | Page 15,16,17 |
|  | 23b | Discuss any limitations of the evidence included in the review. | Page 18,19 |
|  | 23c | Discuss any limitations of the review processes used. | Page 18 |
|  | 23d | Discuss implications of the results for practice, policy, and future research. | Page 17 |
| **OTHER INFORMATION** | | | |
| Registration and protocol | 24a | Provide registration information for the review, including register name and registration number, or state that the review was not registered. | Page 7, subsection 2.1 |
|  | 24b | Indicate where the review protocol can be accessed, or state that a protocol was not prepared. | Page 7, subsection 2.1 |
|  | 24c | Describe and explain any amendments to information provided at registration or in the protocol. | Page 7, subsection 2.1 |
| Support | 25 | Describe sources of financial or non-financial support for the review, and the role of the funders or sponsors in the review. | Page 23 |
| Competing interests | 26 | Declare any competing interests of review authors. | Page 23 |
| Availability of data, code, and other materials | 27 | Report which of the following are publicly available and where they can be found template data collection forms; data extracted from included studies; data used for all analyses; analytic code; any other materials used in the review. | Page 23 |

**Table S2:** Search strategy and literature search results.

| **Database** | **Filter** | **Access Date** | **Search Strategy** | **No of Results** |
| --- | --- | --- | --- | --- |
| **PubMed** | Title/Abstract | 31 July, 2025 | ("SGLT2 Inhibitor" OR "Sodium-Glucose Cotransporter 2 Inhibitor" OR "SGLT2I" OR "Sodium Glucose Cotransporter 2 Inhibitor" OR "Canagliflozin" OR "Dapagliflozin" OR "Empagliflozin") AND (stop OR withhold OR discontinu* OR withdrawal OR withdraw OR use OR manage OR cessation OR interruption) AND (preoperative OR postoperative OR perioperative OR surgery OR operative OR "per-operative" OR "post-operative ") AND (DKA OR "Diabetic Ketoacidosis" OR "metabolic acidosis" OR ketoacid* OR acidosis) | 74 |
| **Cochrane** | Title Abstract Keyword | 31 July, 2025 |  | 20 |
| **Scopus** | Article title, Abstract, Keywords | 31 July, 2025 |  | 88 |
| **WOS** | Topic | 31 July, 2025 |  | 103 |
| **Total** | | | | 285 |

| **Figure S1.** Risk of bias assessment of included studies using the Risk Of Bias In Non-randomized Studies of Interventions (ROBINS-I) tool. |
| --- |
| 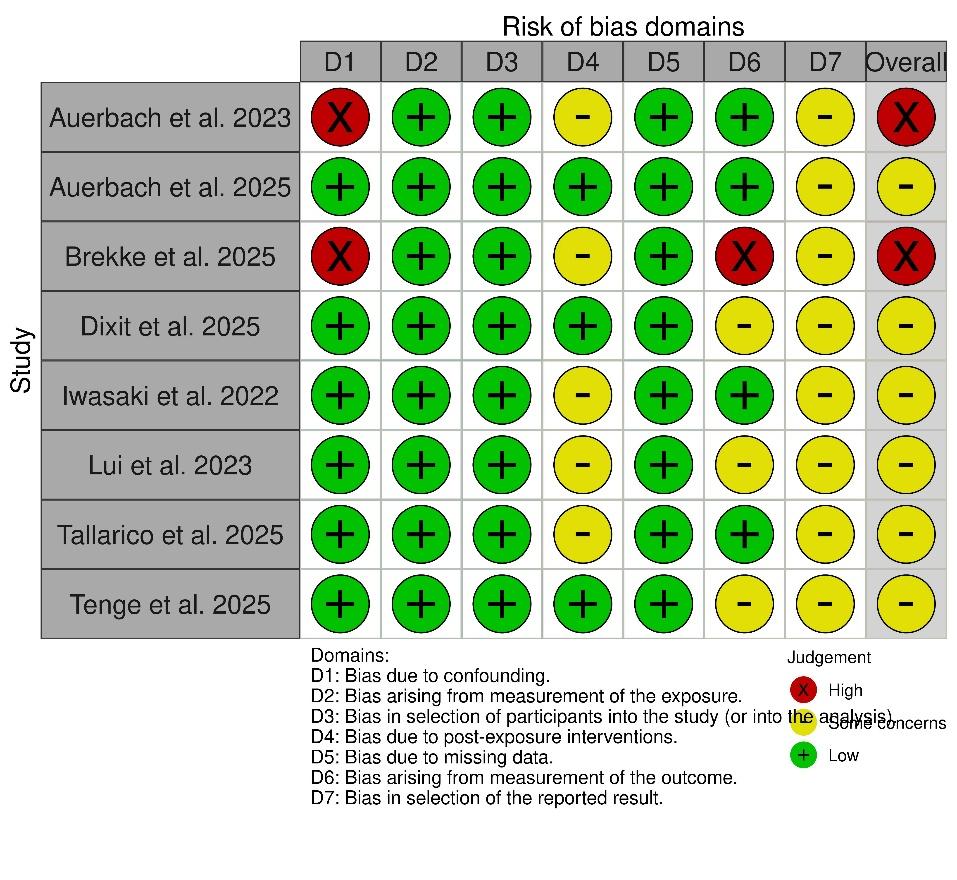 |
| **(A)** |
| 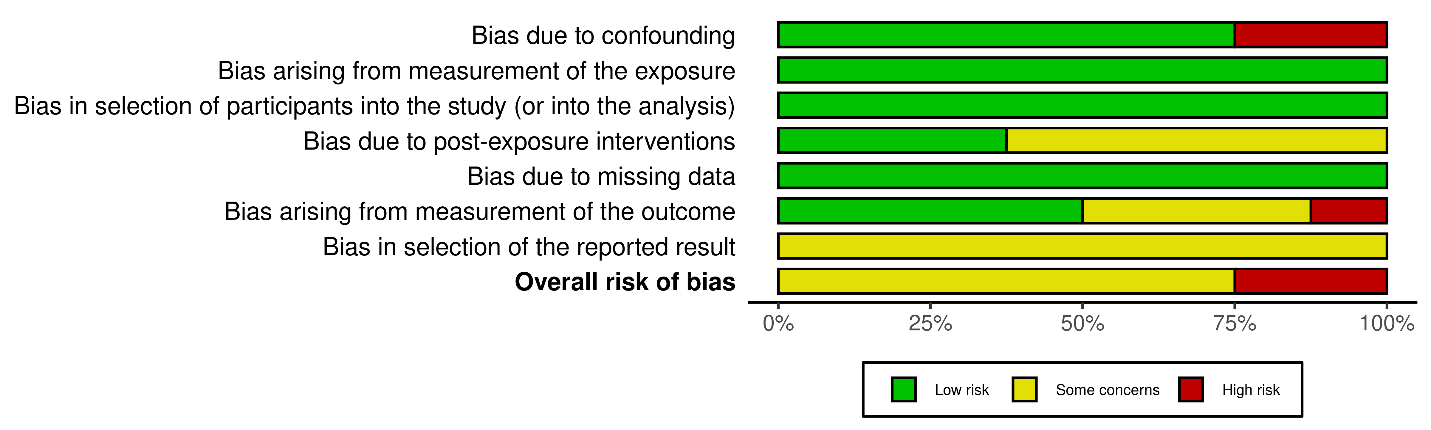 |
| **(B)** |
| **)A(** presents the risk of bias judgments across individual studies and domains. Each domain was assessed as low risk (green), some concerns (yellow), or high risk (red). Overall risk of bias was judged based on the highest domain-level concern. **(B)** summarizes the proportion of studies rated at each risk level across all domains. |

| **Figure S2.** Risk of bias assessment of included randomized controlled trials using the Risk of Bias 2 tool (ROB-2). |
| --- |
| 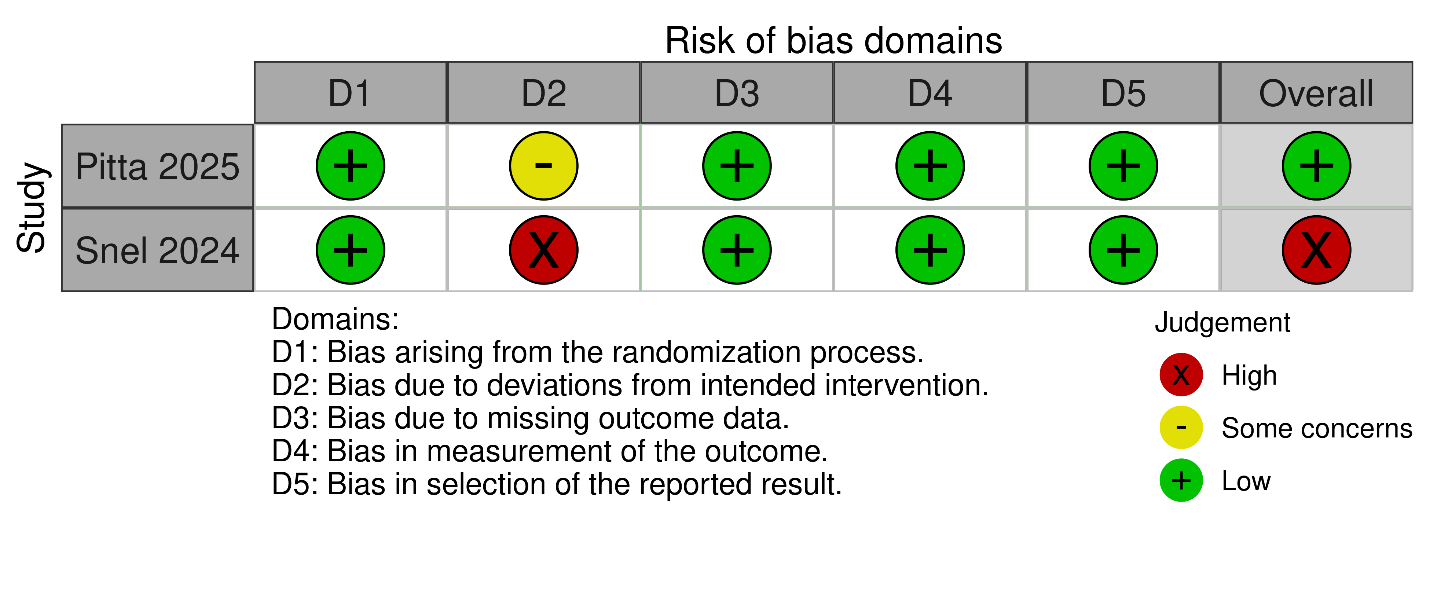 |
| **(A)** |
| 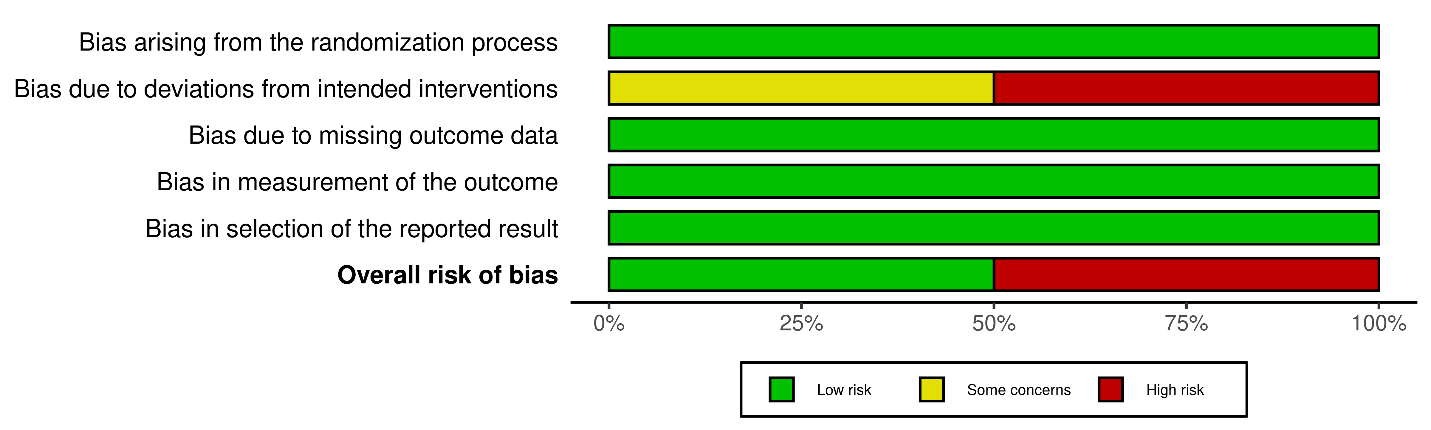 |
| **(B)** |
| **(A)** presents the domain-level judgments for each included randomized controlled trial. **(B)** shows the proportion of studies rated as low risk (green), some concerns (yellow), or high risk (red) across the five bias domains and the overall risk of bias. |


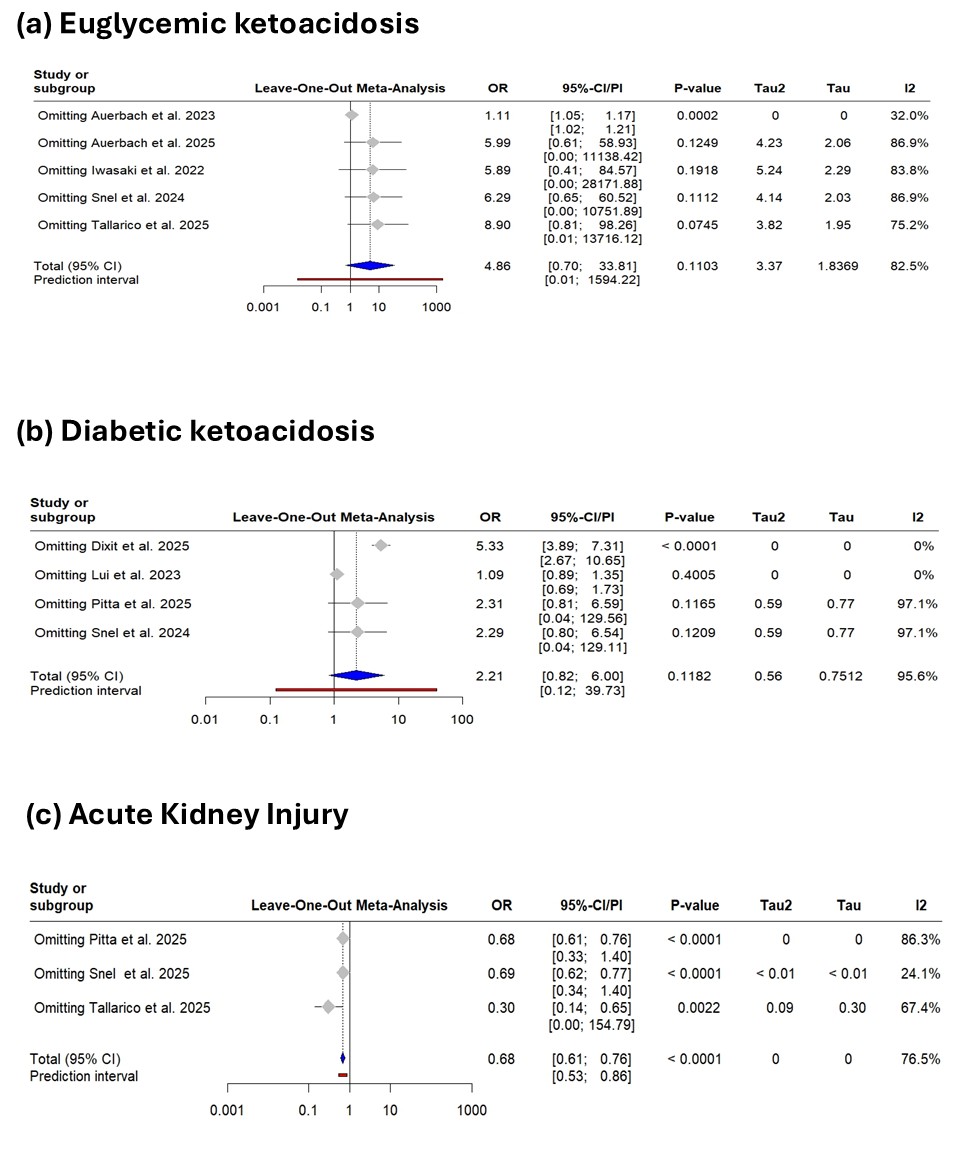


**Figure S3.** Leave-one-out sensitivity analyses for key postoperative outcomes. Panels show the pooled risk ratio with 95% confidence interval (CI) for (a) euglycemic diabetic ketoacidosis, (b) diabetic ketoacidosis, and (c) AKI.

**Figure S3.** Leave-one-out sensitivity analyses for key primary outcomes. Panels show the pooled odds ratio with 95% confidence interval (CI) for (a) euglycemic ketoacidosis, (b) diabetic ketoacidosis, and (c) AKI.


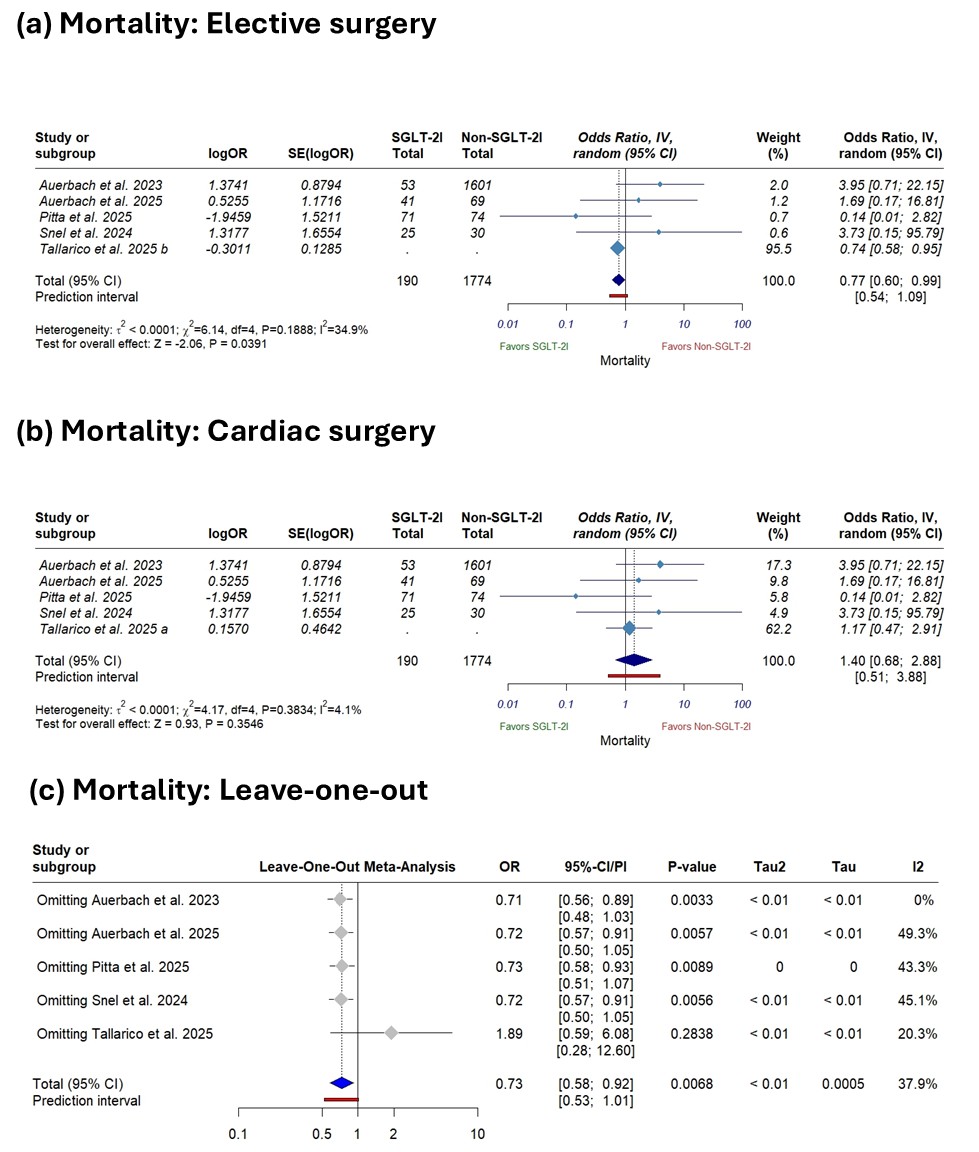


**Figure S4.** Sensitivity analyses for mortality. Panels show the pooled odds ratio with 95% confidence interval (CI) for (a) mortality restricted to elective surgery, (b) mortality restricted to cardiac surgery, and (c) leave-one-out sensitivity.


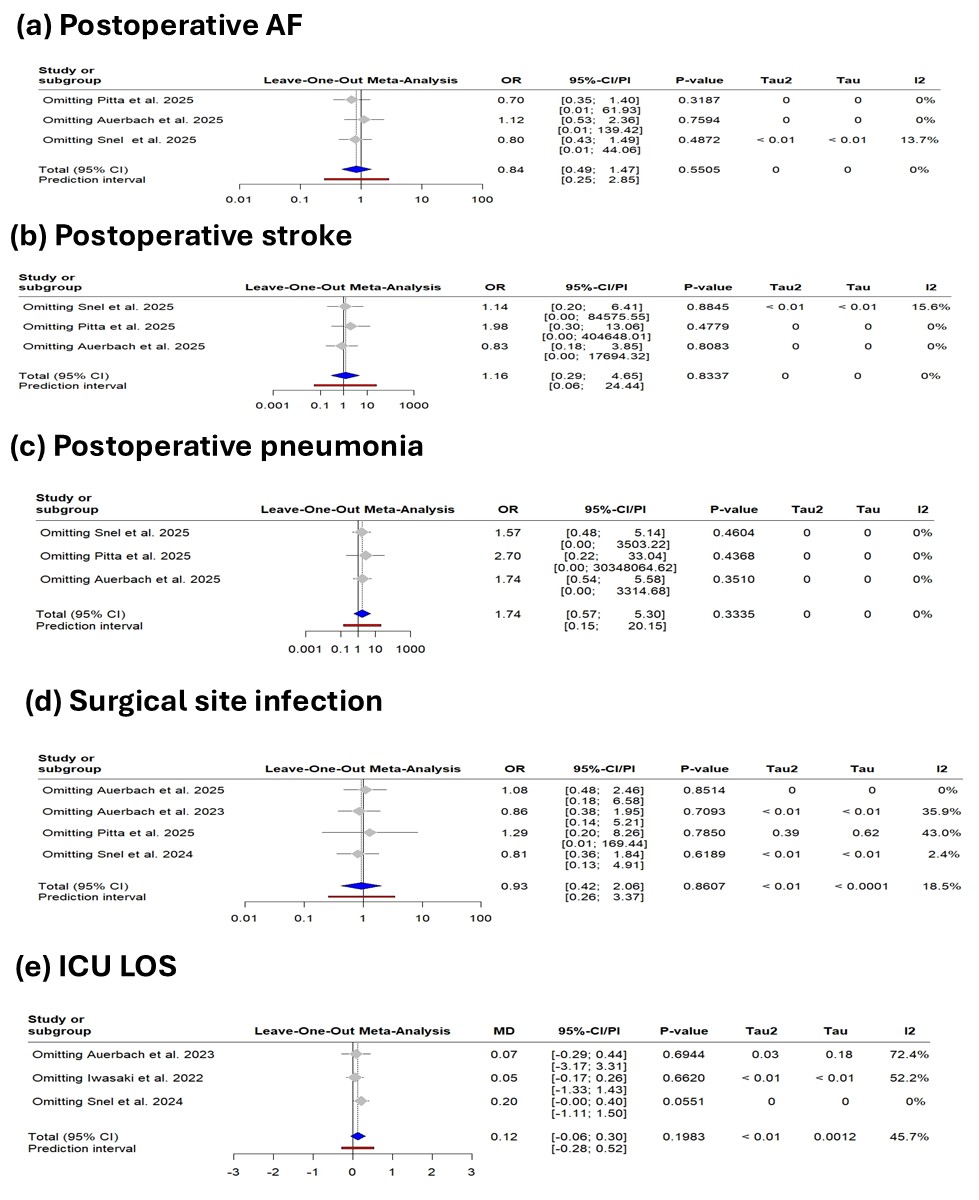


**Figure S5.**  Sensitivity analyses for key secondary outcomes. Panels show the pooled odds ratio with 95% confidence interval (CI) for (a) postoperative AF, (b) postoperative stroke, (c) postoperative pneumonia, (d) surgical site infection, and (e) surgical site infection.


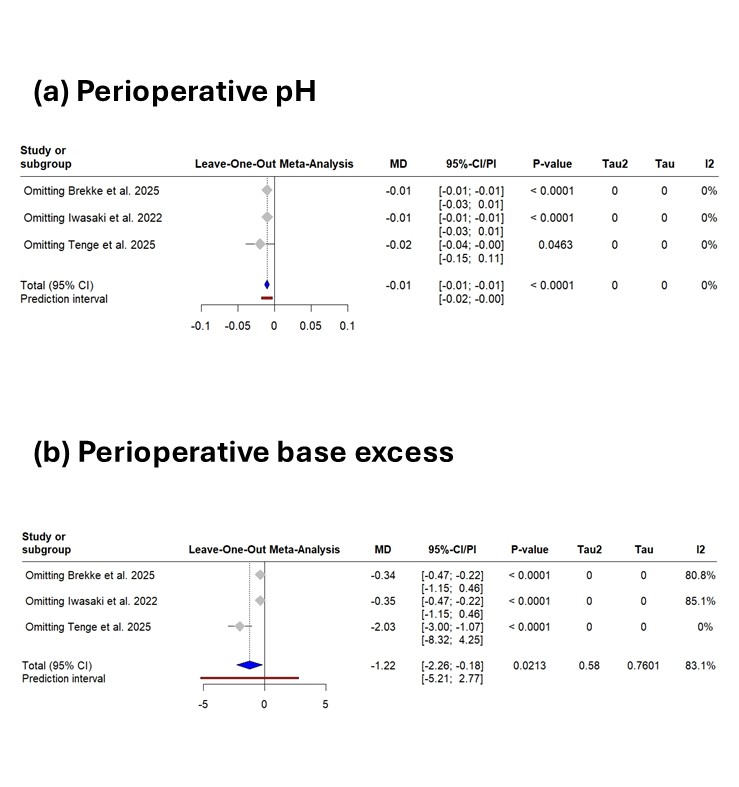


**Figure S6.**  Sensitivity analyses for key laboratory outcomes. Panels show the pooled odds ratio with 95% confidence interval (CI) for (a) perioperative pH and (b) perioperative base excess.
